# Understanding economic openness: a review of existing measures

**DOI:** 10.1007/s10290-020-00391-1

**Published:** 2020-09-01

**Authors:** Claudius Gräbner, Philipp Heimberger, Jakob Kapeller, Florian Springholz

**Affiliations:** 1 Institute for the Comprehensive Analysis of the Economy (ICAE), Johannes Kepler University Linz, Linz, Austria; 2 Institute for Socio-Economics, University of Duisburg-Essen, Duisburg, Germany; 3 Vienna Institute for International Economic Studies (wiiw), Vienna, Austria

**Keywords:** Economic openness, Trade openness, Financial openness, Globalization, F00, F40, F60

## Abstract

This paper surveys measures of economic openness, the latter being understood as the degree to which non-domestic actors can or do participate in a domestic economy. Based on the existing literature, the authors introduce a typology of openness indicators, which distinguishes between ‘real’ and ‘financial’ openness as well as ‘de-facto’ and ‘de-jure’ measures of openness. They use data collected on these indicators to analyze trends in openness over time and to conduct a correlation analysis across indicators. Finally, they illustrate the potential consequences of employing different openness measures in a growth regression framework.

**Electronic supplementary material:**

The online version of this article (10.1007/s10290-020-00391-1) contains supplementary material, which is available to authorized users.

## Introduction

The impact of economic openness on domestic economies has been a prime area of interest within both the scientific community as well as the wider public. The relevant debates, however, use a great diversity of concepts to describe the extent of international economic integration: terms like ‘trade openness’, ‘economic integration’, ‘trade liberalization’ and ‘globalization’ are widely used when the general increase in economic openness during the last decades is addressed. The same observation holds true for the financial dimension, where terms like ‘financial openness’, ‘financial integration’ and ‘financial globalization’ are used regularly and often interchangeably (e.g., Kose et al. 2009; De Nicolo and Juvenal 2014; Saadma and Steiner 2016).

In analogy to this variety of terms and concepts, a large variety of *measures* of economic openness have been developed. These measures typically emphasize different aspects of economic integration. As a consequence, not only the definition, but also the measurement of openness has varied considerably over the past three decades and a corresponding lack of consensus on how to best measure economic openness has been widely acknowledged (e.g. Yanikkaya 2003; Squalli and Wilson 2011; Busse and Koeniger 2012; Huchet-Bourdon et al. 2017; Egger et al. 2019). At the same time, many econometric studies discount the underlying debate on the measurement of economic openness by simply employing the most popular measures without any in-depth explanations or justifications for doing so. Against this backdrop, this paper contributes to the literature by providing a systematic collection, categorization and evaluation of the most prominent openness indicators used in the relevant literature. The main purpose of our work is threefold: first, we provide applied researchers with relevant information to make an informed choice on the use of different openness indicators, which eventually depends on the specific questions and methods employed in their empirical work. Second, we highlight the practical implications of choosing some openness indicator by showing how empirical outcomes change when different openness indicators are used. Third, we compile a data set on openness indicators to be used in further research, where the data are based on 216 countries over the time period 1960–2019, although coverage for individual openness variables varies widely in the country and time dimension. Researcher can access this data set via an openly available R package through which we are able to provide regular updates of the data discussed in this paper, including references to all the primary sources for the relevant indicators.^1^.

In this context, we will operate under two restrictions: first, we consider only measures that are available over a time period of at least 20 years. Second, we restrict ourselves to direct measures of economic openness. As a consequence, we exclude instrumental variables that are sometimes developed to deal with endogeneity problems and to estimate causal effects of openness indicators on outcome measures such as economic growth,^2^ as well as indicators based on extensive models of domestic economies (e.g. Waugh and Ravikumar 2016). While these approaches deserve their own assessment, we confine ourselves to direct measures of economic openness for two main reasons: first, finding a suitable instrument or model capturing trade openness is heavily context-dependent and requires additional theoretical assumptions (e.g. exclusion restrictions). Thus, a *general* assessment of such instruments seems difficult to undertake. Second, direct openness measures as discussed below are not only a prerequisite for instrument design, but also predominant in most of the applied literature (e.g. Dreher et al. 2010; Martens et al. 2015; Potrafke 2015).

The paper proceeds as follows: the next section introduces a typology for openness indicators by discussing the distinction between ‘trade’ and ‘financial’ openness, which have a ‘de-facto’ and ‘de-jure’ dimension, respectively. We classify the most commonly used openness measures according to this typology. Section 3 provides descriptive trends of the most relevant openness indicators, while Sect. 4 analyzes the relationship of these indicators by inspecting the correlations between different openness measures. Section 5 highlights the practical implications of choosing among different measures in a growth regression framework. Section 6 summarizes and concludes the paper.

## Measures of economic openness

Economic globalization and openness are often used interchangeably. In the relevant literature, however, openness is the most common term for capturing phenomena of increasing international integration in trade and finance, and we prefer using it to the term “globalization”. Existing measures of economic openness, generally understood as the degree to which non-domestic actors can or do participate in a domestic economy, can be grouped in two ways: first, according to the type of openness—‘real’ or ‘financial’—they aim to measure, and, second, according to the sources utilized in composing the openness measure. These sources are either aggregate economic statistics (de-facto measures) or assessments of the institutional foundations of economic openness, i.e. the legally established barriers to trade and financial transactions (de-jure measures).

In addition, ‘hybrid’ measures aim to incorporate information on both, real and financial aspects, while “combined” measures also strive to integrate information on de-facto as well as de-jure aspects of economic openness (see Table 1).

**Table 1:** Types of openness indicators

	Evaluation of openness with regard to real flows (goods and services)	Evaluation of openness with regard to financial flows	Combined measures
Evaluation of outcomes: de-facto measures of economic openness	De facto measures of trade openness, for example: *total imports* or *total exports (relative to GDP)*	De facto measures of financial openness, for example: *FDI inward/outward* or *foreign financial assets/liabilities*	Measures integrating real and financial aspects
Evaluation of legal framework: de-jure measures of economic openness	De-jure measures of trade openness, for example: *tariff rates* or *non*-*tariff trade barriers*	De-jure measures of financial openness, for example: *FDI restrictions* or *capital account restrictions*	

De facto measures are outcome-oriented indicators, reflecting a country’s actual degree of integration into the world economy. De-jure measures, on the other hand, are based upon an evaluation of a country’s legal framework: they reflect a country’s willingness to be open as expressed by the prevailing regulatory environment. Typically, de-jure measures on trade are based on tariff rates (such as duties and surcharges), information on non-tariff trade barriers (such as licensing rules and quotas) or tax revenues emerging from trade activities relative to GDP. Financial de-jure measures indicate the extent to which a country imposes legal restrictions on its cross-border capital transactions. As de-jure indicators evaluate a country’s regulatory environment, it is important to keep in mind that this environment is influenced not only by national policies; they are also shaped by the impact of supranational institutions like the European Union or the World Trade Organization.

The above construction and interpretation of the two main types of indicators, de-facto and de-jure, reveals that these types do indeed measure different facets of openness, which need not be consistent for a given country. For instance, a country could have a defensive legal stance in terms of openness, but still play an important role in the world trading system e.g. due to its special position as a trade hub (e.g. China) or as a financial hub (e.g. Malta). At the same time, a country may be open to trade in terms of institutions and policy, but nonetheless lag behind in terms of its real integration in international trade due its geographic remoteness (e.g. Canada) or technological inferiority (e.g. Uganda).^3^

Hence, implications drawn from de-jure indicators can differ strongly from those derived from de-facto indicators as the former are mostly based on a single, yet prominent, factor in shaping actual economic integration—a country’s regulatory environment, while de-facto indicators are focused on overall outcomes. Thus, they capture the *total* impact of a series of different factors, such as the level of technology, geographical location, the existence of natural resources, legal regulations and tax policies, political and historical relationships, multi- and bilateral agreements or the quality of institutions. Therefore, de-facto measures can be seen as capturing the overall impact of all relevant factors without any ambition to delineate their relative contribution to the chosen outcome dimension. It is for these reasons that any “combined measure” (Table 1) has to be received with great care as it lumps together two qualitatively different approaches towards economic openness and can, hence, lead to ambiguous results with unclear interpretations (Martens et al. 2015).

### Trade openness measures

De facto openness to trade in goods and services is a prime subject of interest in discussions on economic openness. The core measure in these discussions is *Trade volume relative to GDP* (Fuji 2019). As Table 1 shows, alternative de-facto openness measures are mostly based on sub-components and variations of the Trade/GDP approach.

The popularity of Trade to GDP probably stems from its availability and its seemingly close alignment to the question at stake. There are also a number of variants, such as exports/GDP or imports/GDP, which can be worthwhile substitutes if one wants to focus on openness understood in either a more ‘outward’ (Exports) or a more ‘inward’ sense (Imports), or restrictions of what enters the numerator, such as variants considering solely trade in goods or excluding exports in primary sectors.

However, despite its popularity Trade/GDP and its variants have to be used with caution for a number of reasons, most of them relating to the normalization by GDP.

First, by taking GDP as a reference point, Trade/GDP incorporates a specific size bias as small economies typically show higher trade volumes relative to GDP than large economies—a fact well-known from the estimation of gravity equations (e.g. Feenstra 2015). As a consequence, strong domestic economies, which also happen to be major players in international trade (like the U.S., Japan, Germany or China), find themselves at the lower end of any country-ranking composed out of Trade/GDP.

Second, it is not entirely clear what Trade/GDP is actually measuring. Various alternatives to the label ‘trade openness’, such as *trade dependency ratio, trade openness index*, *trade share* or *trade ratio*, have been suggested. More recently, Fuji (2019) has discussed this question in greater detail. By comparing values for Trade/GDP for international and intra-Japanese trade data on the prefecture-level, he finds that Trade/GDP measures most of all the extent of spatial economic remoteness and the idiosyncrasy in sectoral production distributions. He also finds that on the international level, much of the variation of the measure goes back to variation in GDP, rather than the trade flows. And indeed, because of the normalization by GDP the Trade/GDP measure also captures cyclical swings of economies.^4^ For instance, the financial crisis in 2008/09 made several countries look ‘more open’ in terms of Trade/GDP, simply because of the disproportionate effect of the crisis on GDP.

Finally, the inclusion of Trade/GDP in regression approaches has also been the target of endogeneity concerns (e.g. Frankel and Romer 2000). Hence, empirical researchers are well-advised to think critically about possible endogeneity problems, especially when coupling Trade/GDP with other GDP-related variables in applied work.

At least the size bias of Trade/GDP has been addressed by various authors, leading to a couple of alternative indicators (see Table 1). Additional strategies for addressing this size-bias include the incorporation of an inversed Herfindahl-Index of the relative shares of all trading partners (to account for the diversity of exchange relations) or regression-based strategies where Trade/GDP is first regressed on a series of demographical and geographical variables and only the residuals of these regressions are interpreted as a measure for ‘net openness’ conditional on some country characteristics (Lockwood 2004, Vujakovic 2010). Whether such corrective measures are appropriate eventually depends on one’s research question and empirical setup. Alternatively, the size-bias of Trade/GDP can be addressed by substituting the Trade/GDP variable with one of the alternatives listed above or by adding additional regressors aiming to control for country size. But it is also evident that every alternative normalization strategy comes with its own problems, which is why the ‘best’ de-facto measure of trade openness depends on the particular question at hand. In this context, Trade/Population could also be an alternative to Trade/GDP that aims to correct only for country size, but not for average income. However, this final alternative has hardly been employed in the applied economics literature so far.

In contrast to the outcome-orientation of de-facto measures, the focus of de-jure measures typically is on tariff rates and other institutional forms of trade-barriers (see Table 3). Unfortunately, there is a lack of de-jure indices that are both methodologically sound and widely available.

One of the earliest and most influential de-jure measures for trade openness is the index by Sachs and Warner (1995). It is a binary index that classifies a country as closed if it meets at least one out of five criteria relating to tariff rates, non-tariff trade barriers, socialist governance in trade relations and the difference between black market exchange rates and official exchange rates. When used in growth regressions, the index mostly suggests a positive relationship between openness and trade (e.g. Harrison 1996; Wacziarg and Welch 2008; Dollar et al. 2016), yet it has been strongly criticized for its ambiguous criterions and its dichotomous output dimension, which classifies countries as either ‘open’ or ‘closed’ and, hence, does not allow for a more nuanced analysis (Rodriguez and Rodrik 2001).

An alternative to the Sachs–Warner-index is the tariff-based measure as used in an influential paper by Jaumotte et al. (2013), who employ a continuous index based on (1) the ratio of tariff revenue to import value and (2) average unweighted tariff rates. By using this measure, they seek to directly measure the changes in the regulatory framework of countries, which is preferable to the rather crude binary index of Sachs and Warner. Unfortunately, the coverage of the dataset provided by Jaumotte et al. (2013) is limited and the authors base their index on internal data of the IMF implying that replicating or expanding their dataset is a non-trivial exercise.

Two further alternatives are provided by two think-tanks, which are known to promote a (normative) free market agenda: the *Trade Freedom Index,* based on the *Economic Freedom Index* of the Heritage Foundation, covers 182 countries from 1995 until 2019, and the *Freedom to Trade Internationally Index*, which is based on the *Economic Freedom of the World Index* of the Fraser Institute. The latter covers the period between 1970 and 2000 in 5-year intervals and contains yearly data over the period 2000–2017 for 161 countries. Both approaches are composite indices that merge several tariff and non-tariff related variables into a final measure (for details see Table 4). Given the partisan origin of these measures in combination with the observation that the data sources and aggregation methods are relatively opaque (see Table 4 for details), it seems that no strong case for considering these two indicators in econometric research can be made.

Aiming to complement the available data-sources, we developed an additional alternative indicator that closely follows the methodological approach of the tariff-based measures of Jaumotte et al. (2013), but is based on the publicly available World Integrated Trade Solution (WITS) databank of the World Bank. Thus, our indicator is easy to replicate and available for 159 countries over the period 1988 to 2018. We calculate the index as 100 minus the average of (1) the effectively applied tariff rates and (2) the weighted average of the most-favored nation tariff rates. The resulting index is strongly correlated with the measure of Jaumotte (with a Pearson coefficient of 0.78 for the joint data points) and, thus, preserves the methodological advantages of the original indicator, while at the same time providing a remedy for its drawbacks in terms of coverage and replicability.

### Financial openness measures

The most popular de-facto measure of financial openness comes from the dataset compiled and continuously updated by Lane and Maria Milesi-Ferretti (2003, 2007, 2017). It is now typically referred to as the “*financial openness index*” and defined as the volume of a country’s foreign assets and liabilities relative to GDP (Baltagi et al. 2009). The Lane and Milesi-Ferretti (henceforth LMF) database is publicly available^5^ and currently contains data for 203 countries for the period 1970–2015. The LMF database is considered the most comprehensive source of information in terms of financial capital stocks. In addition to the financial openness index, this dataset also contains three more specific indicators focusing on FDI and equity markets that have been widely applied in empirical analyses. A comparable set of indicators on FDI can also be obtained from UNCTAD^6^ (see Table 5). It is worth mentioning that these indicators are often normalized by GDP and are, therefore, subject to the same criticisms as the de-facto trade openness measures discussed in Sect. 2.1 (see also Gygli et al. 2019). They are, however, also available in absolute dollar amounts.

Saadma and Steiner (2016) build on the data provided by Lane and Milesi-Ferretti to create an index for private financial openness (OPEN_pv), which can be seen as further development of the financial openness index. It distinguishes between private and state-led financial openness by subtracting development aid (DA) from foreign liabilities (FL) and international reserves (IR) from foreign assets (FA). The motivation of Saadma and Steiner (2016) is to show that correlations between growth and financial openness lead to less ambiguous results when the factors underlying actual capital flows are accounted for in the data.

Finally, Table 6 collects the most prominent de-jure indicators in the financial dimension. Three aspects are of particular importance. First, the IMF’s Annual Report on Exchange Arrangements and Exchange Restrictions (AREAR) plays a prominent role as these reports serve as a key source for deriving de-jure indicators regarding trade openness (IMF 2016).^7^ From this, we can distinguish three sub-categories of financial de-jure measures: (i) de-jure indicators that are based on the AREAER Categorical Table of Restrictions, (ii) de-jure indicators that are based on the actual text of the AREAER and (iii) de-jure indicators that are not based on the AREAER report (Quinn et al. 2011). Table-based indicators provide comprised data and come with the advantage that they are relatively easy to replicate. In contrast, text-based indicators contain finer-grained information on regulatory restrictions of capital flows. As a consequence, text-coded indicators can only be replicated if the authors provide a detailed description of their coding-methodology.

Second, the Chinn–Ito index (KAOPEN) has been widely used in the literature on the impacts of financial openness. It focuses on regulatory restrictions of capital account transactions, is publicly available and covers 181 countries over the period 1970–2017.^8^ This comparably extensive coverage of the Chinn–Ito Index is a major reason for its popularity. The index is based on information about the restrictions on cross-border financial transactions, as provided in the summary tables of the IMF AREAER report (Chinn and Ito 2006, 2008). To compose the index, Chinn and Ito (2008) codify binary variables for the four major categories reported in the AREAR, i.e., (1) the presence of multiple exchange rates, (2) restrictions on current account transactions, (3) restrictions on capital account transactions and (4) the requirement of the surrender of export proceeds. Eventually the KAOPEN index (short for capital account openness index) is constructed by conducting a principal component analysis on these four variables.^9^

### Hybrid and combined measures for economic openness

While there is a number of different indicators for assessing the intensity of globalization in general (see Gygli et al. 2019, Table 2, for an overview), indices that focus specifically on *economic* globalization (as distinguished from e.g. social, political or cultural aspects of globalization) are comparably rare. To derive such more specific measures of economic globalization requires researchers to first isolate the relevant economic dimensions and then identify suitable variables for measuring these dimensions. Among those globalization indicators that could serve as a starting point for assessing the economic dimension of globalization—such as the DHL Connectedness index (Ghemawat and Altman 2016), the New Globalization index (Vujakovic 2010), or the Maastricht Globalization index (Figge and Martens 2014)—the KOF Globalization index (Dreher 2006; Gygli et al. 2019) occupies an exceptional position in terms of coverage, conceptual clarity and transparency. The index is supplied by the Swiss Economic Institute (KOF) and is by far the most widely applied index of economic openness in the economics literature (Potrafke 2015). Most recently, the KOF introduced a series of methodological improvements as well as additional variables to revise and extend the basic methodology for constructing the KOF globalization index (Gygli et al. 2019). In doing so, the KOF also introduced a series of novel sub-indices based on a modular structure, which allows for inspecting different dimensions of economic openness in a disaggregated form.

**Table 2:** De-facto trade openness measures

Name	Components	Scale	Type	Time	Countries	Source
Export share	Exports (X)	% of nominal GDP	Co-Ra	1960–2018	199	World Bank (2017) (publicly available)
Import share	Imports (M)		Co-Ra			
Trade share	Trade Volume = Exports (X) + Imports (M)		Co-Ra			
Generalized trade openness index	The Index represents the trade volume as a share of a country’s GDP factor, defined by a CES-function of its own GDP and the GDP of the rest of the world	0–100	Co-Int	1960–2016	167	Tang (2011) (own calculations)
composite trade share	Trade Volume (X + M) in % GDP, adjusted by the World Trade Share (WTS)	arbitrary	Co-Int	1977–2016	231	Squalli and Wilson (2011) (own calculations)
Real trade share	Trade Volume (X + M) in % of GDP at PPP	% of real GDP	Co-Ra	1960–2014	173	Alcala and Ciccone (2004) (own calculations)
Adjusted trade share	Imports divided by GDP, adjusted for the nation’s share in world production	arbitrary	Co-Ra	1960–2016	233	Li et al. (2004) (own calculations)

In the type column “Co” corresponds to “continuous”, “Int” corresponds to “interval”, and “Ra” corresponds to “Ratio”.

## Descriptive statistics for the openness indicators

This section illustrates some of the general trends and properties exhibited by the indicators presented so far.

### Trade openness

**Fig. 1:**
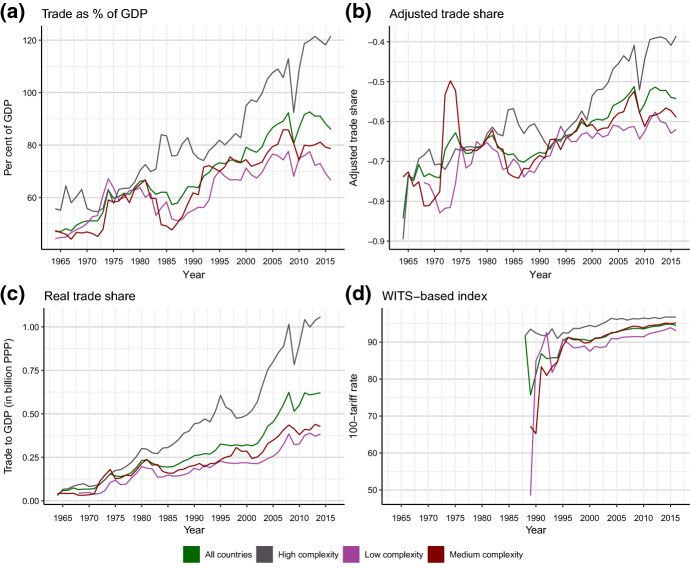
Trends of trade indicators (panels **a**–**c** show de-facto measures; panel **d** a de-jure measure). Sources: See Tables 2 and 3

Panels A and B in Fig. 1 show descriptive statistics of selected trade indicators. We classify countries according to their economic complexity (Hidalgo and Hausmann 2009), a proxy for the level of their technological capabilities.^10^ This is motivated by findings according to which countries with high economic complexity tend to benefit more from trade (e.g. Carlin et al. 2001; Hausmann et al. 2007; Huchet-Bourdon et al. 2017). Indeed, we observe some substantial differences in de-facto trade openness when considering technological capabilities. Specifically, we find that the trade-to-GDP ratio of high complexity countries started to decouple from the moderate and low complexity countries in the early 1990s.^11^ This finding suggests that countries that are technologically superior (and are, thus, likely to benefit more from trade) tend to record higher de-facto openness to trade. We can also see trade integration has reached a peak before the start of the financial crisis in 2007/2008 according to the de-facto trade openness dimension. Against the background of changes in trade policy—in particular in the case of the US under president Trump (e.g. Eichengreen 2018)—and the potential repercussions of the COVID-19 crisis on the globalization process, de-facto international trade integration may be expected to continuously proceed at a much slower space than in earlier decades.

With regard to de-jure openness to trade, the differences across country groups are less pronounced than in the de-facto dimension, as we see convergence since the late 1980s (Fig. 1, panel d). The latter observation suggests that countries of moderate and low complexity have opened their regimes in terms of trade policy in the past decades and all countries have approached high degrees of de-jure openness. Several factors have been discussed in the literature to explain this change in trade policy (especially in developing countries), ranging from the policy-makers’ intention to increase trade volumes to the effects of trade agreements within the WTO and policy prescriptions advocated by the IMF and the World Bank (e.g. Baldwin 2016; Rodrik 2018).

### Financial openness

**Fig. 2:**
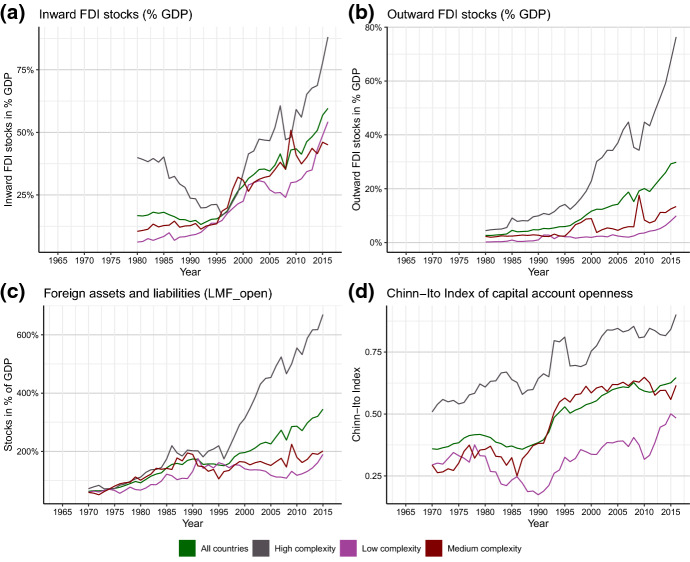
Trends in indicators for financial openness (panels **a**–**c** show de-facto measures; panel **d** a de-jure measure). Sources: See Tables 5 and 6

Compared to trade openness, measures of financial openness show a similar, but even stronger trend (see Fig. 2, panels a–d). De facto measures of the high complexity group started to decouple from the other groups between 1995 and 2000, that is, after the foundation of the WTO in 1994. Since then, the gap between the former and the latter two groups has grown substantially, which implies that financial integration among high complexity countries has proceeded faster than in the rest of the world. The large outward FDI stock of high complexity countries indicates that a large part of FDI in medium- and low complexity countries, where inward FDI is much larger than outward FDI, stems from the high complexity country group. Eventually, we observe that the financial crisis of 2007–08 only had a minor impact on financial openness: after a sharp reduction, the level of financial de-facto openness recovered rapidly and continued to grow across all country groups, which has not been the case for de-facto trade openness.

In terms of financial de-jure openness, we find that high complexity countries have kept the high level of financial de-jure openness established during the 1990s constant over the past two decades. In contrast, countries with moderate and low complexity have seen their de-jure openness increase till the advent of the financial crisis in 2007/2008—since then, the Chinn–Ito index (Fig. 2, panel d), which is the only index covering all years in the time-span of interest, indicates that financial openness in medium complexity countries has decreased, while it has increased in low complexity countries.

**Fig. 3:**
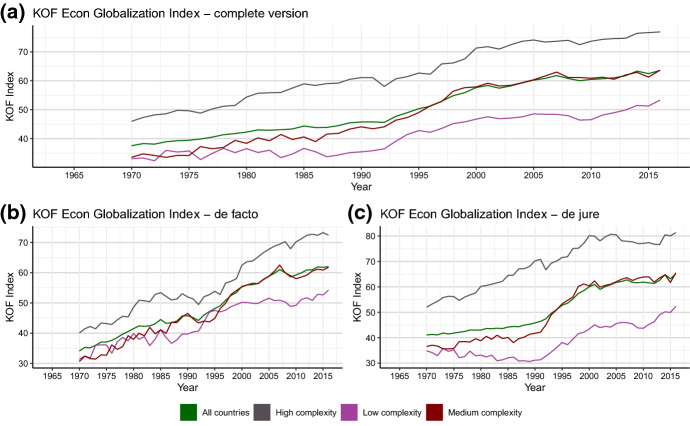
The KOF globalization index as a hybrid measure. Source: see Table 7

The KOF index provides a more complete view on the increase of economic openness in the previous decades and the plateauing of the economic globalization process since the global financial crisis. As can be seen from Fig. 3, the index captures the overall trend of increasing openness from the 1970s to the 2000s (plot A) and mimics the somehow different dynamics in the de-facto and de-jure dimension (plots B and C). In the de-facto dimension, the KOF-index clearly depicts the on-going divergence in terms of economic openness between high complexity countries and the rest of the world, which has already been visible in Figs. 1 and 2. Similarly, the weak but persistent trend for a convergence in terms of the de-jure openness is picked up by the KOF-index. From a global perspective, the main increase in de-jure openness happened in the 1990s, in which all three country-groups, on average, experienced a significant increase in de-jure openness.

## Do different measures of openness agree? A correlation analysis

After introducing the most prominent indicators for economic openness and discussing their conceptual differences, we will now examine the empirical relationship between these openness indicators. Given the previous discussion, we would expect that indicators within the same group (e.g. de-facto trade openness) measure similar aspects of economic openness and, therefore, are strongly correlated with each other. To corroborate this hypothesis and to study the relationship between indicators belonging to different types, we now conduct a comprehensive correlation analysis of all available openness indicators (as well as their specific sub-components and variants) presented so far, which are technically suitable for such an analysis.

**Fig. 4:**
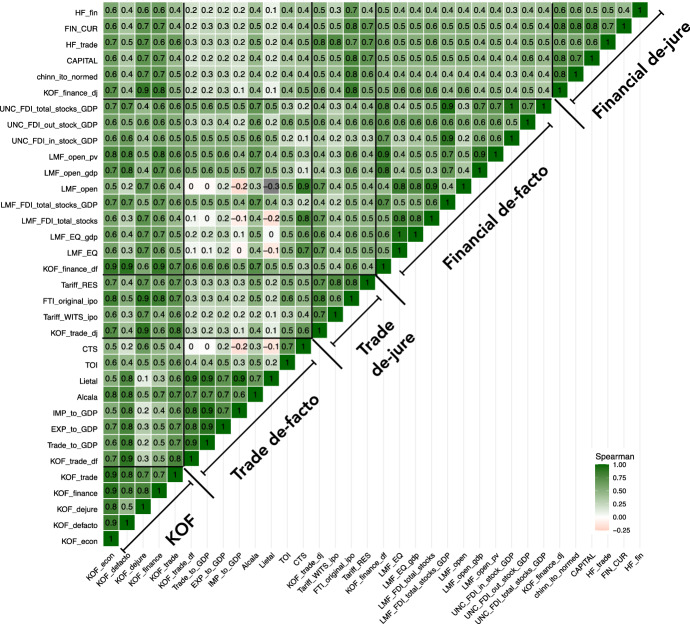
Spearman correlation coefficients for the levels of the openness indicators discussed in this paper. Sources: see Tables 2 to 7; own calculations

**Fig. 5:**
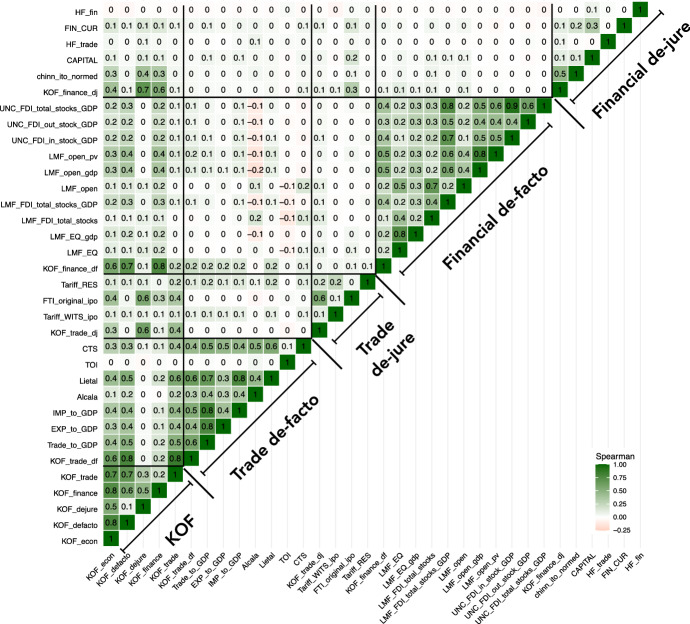
Spearman correlation coefficients for the first differences of the openness indicators discussed in this paper. Sources: see Tables 2 to 7; own calculations

Since many papers use the first difference of these indicators, we pay attention to both, correlations of the variables in levels as well as in first differences.^12^ This exercise is useful for answering a variety of questions: for instance, whether indicators that were built to measure the same type of openness are consistent with each other or to what extent financial and trade indicators do behave similarly. In addition, such an approach allows for clarifying the degree of alignment between one-dimensional indicators on the one hand and hybrid and combined indicators on the other hand. Finally, studying the relationship between different indicators is a relevant preliminary exercise for examining the question whether the choice of indicators matters for empirical applications. In our analysis, we use the Spearman rank coefficient since it requires only few assumptions on the scale and distribution of the compared time-series (e.g. Weaver et al. 2017). We report the results using the Pearson coefficient, which are qualitatively very similar, in the accompanying appendix. While Fig. 4 illustrates the correlation of the various measures in levels, Fig. 5 depicts correlations among the time series of the various indicators in first differences. The correlation analysis is based on 216 countries from 1965 to 2019, but for the individual indicators there are restrictions in the underlying country and time periods (see Tables 2, 3, 4, 5, 6 and 7). Given these data restrictions, we calculate pair-wise correlations.

**Table 3:** De-jure trade openness measures

Name	Components	Scale	Type	Time	Countries	Source
Sachs–Warner index	Binary variable based on Sachs and Warner (1995) criterion (see text for more details)	0–1	Di-Bi	1960–2010	118	Sachs and Warner (1995) Extended by Wacziarg and Welch (2008) and Dollar et al. (2016) (publicly available)
IMF tariff rates (Tariff_RES)	100—average of the effective rate (= tariff revenue/import value) and the average unweighted tariff rates	0–100	Co-Int	1980–2005	136	Jaumotte et al. (2013), based on IMF database (publicly available)
Trade freedom (HF_trade)	Trade-weighted average tariff rate—nontariff trade barriers (NTBs)	0–100	Di-Int	1995–2019	182	Miller et al. 2020: Index of Economic Freedom. Heritage Foundation (publicly available)
Freedom to trade internationally (FTI_Index)	1. Tariffs: Revenue from trade taxes (% of trade sector) Mean tariff rate Standard deviation of tariff rates 2. Regulatory trade barriers: Non-tariff trade barriers Compliance costs of importing and exporting	0–10	Co-Int	5-year measure: 1970–2000 Yearly data: 2000–2017	161	Gwartney et al. (2017): economic freedom of the world: 2017 annual report. fraser institute. (publicly available)
*Additional variable with improved coverage*						
WITS tariff rates (Tariff_WITS)	100—mean of effectively applied (AHS) and most-favored nation (MFN) weighted average tariff rates	0–100	Co-Int	1988–2018	159	Based on tariff data of WITS databank (own calculations)

In the type column “Co” corresponds to “continuous”, “Di” corresponds to “discrete”, “Bi” corresponds to “binary”, “Int” corresponds to “interval”, and “Ra” corresponds to “Ratio”

**Table 4:** Components of the trade freedom and the freedom to trade internationally index

Variable	Description	Source and further details
*Trade freedom index*		
$${\text{Trade}}\;{\text{freedom}} = 100 \cdot \frac{{{\text{Tariff}}_{max} - {\text{Tariff}}_{x} }}{{{\text{Tariff}}_{max} - {\text{Tariff}}_{min} }} - NTB$$		
Tariff_X_	Weighted average tariff rate in country X	Miller et al. (2020)
Tariff_max_, Tariff_min_	Upper and lower bounds for tariff rates	
NTB	Minimum tariff is zero, the upper bound is set to 50 percent. Depending on the use of NTBs a penalty is subtracted from the base score	

Variable	Description	Source
*Freedom to trade internationally index*		
$$FTI = \frac{1}{5}\mathop \sum \limits_{n = 1}^{5} \delta_{i}$$		
Tariff dimension		
$\delta_{1}$	Revenue from trade taxes	Fraser Institute (2020)
$\delta_{2}$	Mean tariff rate	
$\delta_{3}$	Standard deviation of tariff rates	
Regulatory trade barriers (included since 1995)		
$\delta_{4}$	Non-tariff trade barriers	
$\delta_{5}$	Compliance costs of importing and exporting

**Table 5:** De-facto financial openness measures

Name	Components	Scale	Type	Time	Countries	Source
Financial openness index (LMF_OPEN_gdp)	LMF_OPEN represents the sum of Total Foreign Assests and Total Foreign Liabilities in % GDP	% of GDP	Co-Ra	1970–2015	203	“LMF”: Lane and Milesi-Ferretti (2017) (publicly available)
Equity-based financial integration (LMF_EQ_gdp)	LMF_EQ represents the sum of Portfolio Equity Assets and Liabilities (stocks)	% of GDP	Co-Ra	1970–2015	203	
Private financial openness index (OPEN_pv)	OPEN_pv makes a distinction between private and official financial openness by subtracting official development aid from foreign liabilities and international reserves from foreign assets.	% of GDP	Co-Ra	1970–2014	179	Saadma and Steiner (2016)
FDI asset stock (UNCTAD) (UNC_FDI_out_stock_GDP, UNC_FDI_in_stock_GDP)	The inward FDI stock represents the value of foreign investors’ equity in and net loans to enterprises resident in the reporting economy The outward FDI stock represents the value of the resident investors’ equity in and net loans to enterprises in foreign economies	% of GDP	Co-Ra	1980–2018	197	UNCTAD (2017) (publicly available)

In the type column: “Co” corresponds to “continuous”, and “Ra” corresponds to “Ratio”

**Table 6:** Classification of financial de-jure measures

Name	Components	Scale	Type	Time	Countries	Source
Chinn–Ito-Index (KAOPEN)	Table-based AREAER* measure: Presence of multiple exchange rates Restrictions on current account transactions Restrictions on capital account transactions the requirement of the surrender of export proceeds	arbitrary	Co-I	1970–2017	181	Chinn and Ito (2006) update in 2015, (publicly available)
Financial Current Account (FIN_CURRENT)	Text-based AREAER* measure FIN_CURRENT is based on how compliant a government is with its obligations under the IMF’s Article VIII to free from government restriction the proceeds from international trade of goods and services	0–100	Di-O	1960–2004	95	Quinn and Toyoda (2008) (publicly available)
Capital Account Liberalization (CAPITAL)	Text-based AREAER* measure CAPITAL is based on restrictions on capital outflows and inflows, with a distinction between residents and non-residents	0–100	Di-O	1960–2004	94	Quinn and Toyoda (2008) (publicly available)
Capital Account Restrictions (KA_Index)	Text-based AREAER* measure Similar than CAPITAL and FIN_CURRENT but includes finer-graned sub-categories and information about different types of restrictions, asset categories, direction of flows and residency of agents.	0–1	Di-O	1995–2005	91	Schindler (2009) (publicly available)
Financial Current and Capital Account (FOI)	Table and text-based AREAER* measure The most comprehensive AREAER* measure. The FOI includes information on twelve categories of current and capital account transactions (more see text)	0–12	Di-O	1965–2004	187	Brune (2006) (not available)
Investment Freedom (HF_fin)	Non-AREAER* measure Index starts from 100 and then points are deducted due to a penalty catalogue. Information based on official country publications, the Economist and US government agencies, but exact coding/methodology remains unclear.	0–100	Di-O	1995–2019	182	Miller et al. (2020), (publicly available)
Equity market liberalization indicator	Non-AREAER* measure This binary liberalization index corresponds to a date of formal regulatory change after which foreign investors officially have the opportunity to invest in domestic equity securities.	0–1	Di-Bi	1980–2006	96	Bekaert et al. (2013) (not available)

In the type column: “Co” corresponds to “continuous”, “Di” corresponds to “discrete”, “Bi” corresponds to “binary”,  “O” corresponds to “ordinal”, “I” corresponds to “interval”

**Table 7:** The KOF economic globalization index as an example for a hybrid measure

Name	Components	Scale	Type	Time	Countries	Source
KOF trade de-facto	Trade in goods (40.9%)	0–100	Co-Int	1970–2017	221	Gygli et al. (2019), (publicly available)
	Trade in services (45%)					
	Trade partner diversification (14.1%)					
KOF finance de-facto	Foreign direct investment (27.5%)					
	Portfolio investment (13.3%)					
	International debt (27.2%)					
	International reserves (2.4%)					
	International income payments (29.6%)					
KOF de-facto	KOF trade de-facto (50%)					
	KOF finance de-facto (50%)					
KOF trade de-jure	Trade regulations (32.5%)					
	Trade taxes (34.5%)					
	Tariffs (33%)					
KOF finance de-jure	Investment restrictions (21.7%)					
	Capital account openness (78.3%)					
KOF de-jure	KOF trade de-jure (50%)					
	KOF finance de-jure (50%)					
KOF econ	KOF de-facto (50%)					
	KOF de-jure (50%)					

Notes: In the type column: “Co” corresponds to “continuous”, “Int” corresponds to “interval”

For more details see: https://ethz.ch/content/dam/ethz/special-interest/dual/kof-dam/documents/Globalization/2019/KOFGI_2019_structure.pdf (accessed April 22nd, 2020)

When inspecting Figs. 4 and 5, we can identify clusters of closely related openness measures: we generally find stronger associations among the indicators within each type (trade de-facto; trade de-jure; financial de-facto; financial de-jure), but only weak to moderate correlations of indicators can be established across different types (e.g. trade de-facto vis-à-vis financial de-facto)—with some notable exceptions to be discussed below. Thereby, correlations are consistently weaker whenever one compares the differenced indicator (Fig. 5), with indicators of different types now being almost completely uncorrelated. Furthermore, these correlations reveal that de-jure measures on trade and financial openness are more closely correlated than its de-facto counterparts, while the correlation between de-facto and de-jure in both dimensions (trade and finance) is weak. This result implies that economic policy in terms of trade and finance tends to be more convergent than de-facto outcomes; furthermore, countries that decide to reduce institutional obstacles to trade generally do it simultaneously for real and financial flows. Our findings lend support to the argument that de-facto indicators generally represent more than just the outcome of economic policy, while de-jure indicators measure the legal foundations of economic policy.

Across the four major types of openness, the cluster relating to de-facto financial openness measures is the least visible cluster, which indicates that this dimension exhibits the greatest diversity in terms of indicators with different conceptual underpinnings. Notably, we find that the KOF economic globalization index is correlated with almost all other indices, which illustrates its ability to integrate different aspects of economic openness.

In sum, the correlation analysis suggests that the concept of ‘economic openness’ has many facets, and various measures capture quite different aspects of this ‘openness’.

## Application: the choice of economic openness measures makes a difference in growth regressions

We continue by posing a question that is of particular interest to empirical researchers: what do the findings from the correlation analysis in the previous section imply for the choice of openness variables in regression specifications? For illustration purposes, we run growth regressions based on a data set for 65 countries over the time period 1995–2014. The choice of this data sample was driven by data restrictions: we only included observations when data for all the different economic openness indicators were available. If we would allow for differences in the data sample used for estimating models with various openness indicators, we would be unable to provide a clear interpretation about whether using different openness measures has an impact on the reported results. Our choice of the data sample, on which we provide more detailed information in the appendix, therefore, facilitates comparative interpretations.

There exists a large literature on the determinants of economic growth (e.g. Barro 1991; Barro and Sala-i-martin 1995; Aghion and Howitt 2008), which has partly focused on the impact of increasing economic openness (e.g. Dollar 1992; Sachs and Warner 1995; Frankel and Romer 2000; Arora and Vamvadikis 2005). While this literature has produced mixed results regarding the link between openness and growth (e.g. Rodriguez and Rodrik 2001; Eichengreen and Leblang 2003; Singh 2010), a number of studies has highlighted that the choice of the openness indicator can have a pronounced impact on the obtained regression results (e.g. Rodriguez and Rodrik 2001; Yanikkaya 2003; Arribas Fernández et al. 2007; Quinn et al. 2011). Against this background, we apply the trade and financial openness indicators analyzed in the first sections of this paper in a standard growth regression framework; by doing so, we illustrate how the choice of the openness variable matters.

Over the last decades, many economists have put forward the argument that economies that are more open to trade grow more quickly. Potrafke (2015, p. 518) puts the dominant prediction in a nutshell: “Globalisation is expected to spur economic growth for many reasons. Trade openness enables, for example, countries to exploit comparative advantages, to gain from specialisation, to foster innovation and efficient production.” When it comes to financial globalisation, the most forceful prediction with the most influence on policy debates has also clearly pointed to overall positive growth effects, especially during the times of the “Washington Consensus” (e.g. Rodrik 2006). Although the theoretical predictions concerning the effect of economic openness on growth can be seen to be less clear-cut on closer inspection, especially when it comes to financial openness (e.g. Stiglitz 2004), this broad theoretical conviction has guided large parts of the econometric literature.

Our regression equation closely follows standard specifications as used in the existing literature (Barro and Sala-i-martin 1995; Arora and Vamvadikis 2005) and can be summarized as follows:

1 $$GDPg_{i,t} = \alpha open_{i,t} + \delta Z_{i,t} + FE_{i} + \varepsilon_{i,t} ,$$

where $GDPg_{i,t}$ represents the growth rate of real GDP per capita for country i in period t. $open_{i,t}$ is the main explanatory variable of interest, defined as the natural logarithm of one of several (trade or financial) openness indicators, which we introduce below. $Z_{i,t}$ represents a vector of additional explanatory variables, which are explained in Table 8 (data sources and summary statistics are available in the accompanying Online Appendix). $FE_{i}$ are country-fixed effects, which we include to account for unobservable, time-invariant country-specific characteristics that may influence $GDPg_{i,t}$. In this setup, we express all variables as non-overlapping 5-year averages (except for the initial level of GDP per capita) to dampen the effects of short-run business cycle fluctuations on GDP per capita growth (e.g. Arora and Vamvadikis 2005). Additionally, and to account for the correlation structure found for the times series in first differences (compare Figs. 4 and 5), we also estimate a corresponding version of Eq. (1) in first differences (FD)^13^:

2 $$\Delta GDPg_{i} = \Delta open_{i,t} \alpha + \Delta Z_{i,t} \delta + \varepsilon_{i,t}$$

**Table 8:** The results from estimating Eqs. (1) and (2) with different measures for economic openness

	Direction of relationship		Significance		Controls
	5-year averages	FD yearly	5-year-averages	FD yearly	
*Dependent variable: GDP per capita growth*					log(human capital), population growth, inflation, log(investment share) *For 5*-*year estimations additionally:* log(initial GDP),
Trade de-facto					
Trade to GDP	+	+	0	**	
Real trade share	+	+	***	0	
Adjusted trade share	+	+	0	0	
Composite trade share	+	+	0	0	
Generalized Trade Openness Index	−	−	0	***	
KOF de-facto	+	−	0	***	
Trade de-jure					
KOF_de-jure	+	+	**	0	
Tariff_WITS	+	−	***	0	
FTI_Index	+	−	0	0	
HF_trade	+	+	0	0	
Financial defacto					
LMF_open	+	−	0	**	
LMF_EQ	−	−	0	***	
FDI inward stocks	+	−	0	***	
FDI outward stocks	+	−	0	0	
Finan. de-jure					
KAOPEN	+	+	0	0	
HF_fin	−	−	0	0	
CAPITAL	+	+	0	**	

We use 5-year averages when estimating Eq. (1) and annual data when estimating Eq. (2). The dependent variable is GDP per capita growth and the openness measures were transformed into natural logarithms. Statistical inference is based on clustered (heteroskedasticity-robust) standard errors. “FD yearly” denotes First Differences based on annual observations

Country- and time-fixed effects were included. When running the specifications in first differences, however, the country-fixed effects drop out algebraically. All models estimated with data based on 65 countries over the period 1995–2014

*, **, and *** denote statistical significance at the 10%, 5% and 1% level, respectively.

The results on the sign and statistical significance of the estimated coefficients are summarized in Table 8. It should again be emphasized that the purpose of the growth regressions is simply to illustrate how using different openness variables can affect the results when we use a consistent data sample, and not to come up with a definitive or comprehensive growth model.

While our specifications will contain misspecifications, most notably due to endogeneity issues, the outcomes reveal interesting patterns, both within and between the various dimensions of openness, and thereby highlight the implications of choosing among different openness measures. Within the cluster of de-facto trade openness measures, and for the case of 5-year averages in levels, the Generalized Trade Openness Index (Tang 2011) suggests a negative relationship between openness and growth. The remaining indicators, on the other hand, suggest a positive relationship, and only the real trade share obtains statistical significance. The picture is more ambiguous when we consider the first-difference estimations based on annual data: in this case, the Generalized Trade Openness Index and the KOF de-facto indicator show a negative sign, but only the latter is statistically significant. The remaining four indicators are positively correlated with growth, with trade to GDP being significantly so. These marked differences in how openness indicators correlate with GDP growth can be traced back to the methodological approach underlying the construction of different openness indicators, which is why our comparison of growth regressions results provides an illustration for the theory-ladenness of observation (Hanson 1958) in the context of measuring economic openness. The fact that moving from one measure for de-facto openness to another has such profound effects on the estimation results—remember that the underlying data sample in the different regressions is the same—strengthens our point that the choice of the indicator is important and requires both a case-based theoretical justification as well as thorough robustness checks.

The results within the cluster of trade de-jure measures are also mixed: in case of the 5-year averages, all indicators (KOF_dejure, Tariff_WITS, HF_trade and the FTI index) are positively correlated with growth, but only the first two variables are statistically significant. The results for the FD-specifications show that the Tariff_WIT and the FTI index coefficients switch signs, although they also remain statistically insignificant.

The conclusion for measures of de-facto financial openness is also ambiguous: in case of the 5-year averages, three of the four de-facto measures suggest a positive relationship (LMF_open, FDI inflows, FDI outflows), with two of them being statistically significant, while one LMF openness indicator (LMF_EQ) has a negative sign. The results are more straightforward when the FD estimator is used: here all indicators suggest a negative relationship and all these correlations, except for the FDI outflows, are considered as statistically significant at the 5% or 1% percent level.

Finally, we also observe ambiguous patterns for the financial de-jure measures with KAOPEN and CAPITAL being positively, and HF_fin being negatively associated with growth, for both the estimations based on first differences and 5-year averages. However, only the CAPITAL coefficient in the FD-case shows statistical significance.

These exercises reveal that there is not only considerable variation in outcomes when different types of economic openness are considered, but that results may also vary within a certain conceptual dimension as different indicators are constructed in different ways. To arrive at a fuller picture of the empirical assessment of economic openness, we estimate a more complete regression equation in the next step. In doing so, we augment the baseline specification by explicitly including measures for different types of economic openness in each single model.

The results regarding the determinants of GDP per capita growth obtained from these estimations are again sensitive to both the dimensions of economic openness actually considered as well as to the set of openness indicators chosen to represent different dimensions of openness (see Table 9). When we only include the KOF_econ indicator, we find a positive but statistically insignificant coefficient regarding the impact of economic openness on growth. However, when separating trade and financial openness using the KOF_trade and KOF_finance subindicators in models (3) and (4), we arrive at a more nuanced result: while the coefficient of KOF_trade is always positive and significant, KOF_finance is negative and in the FD specification statistically significantly so. In models (5) and (6), we find that the KOF de-jure measures (both in the trade and in the finance dimension) generally correlate positively with economic growth, but no such consistent observation is possible for the de-facto measures. The even more disaggregated models (7) and (8) suggest that openness to trade tends to correlate positively with growth in both de-facto and de-jure terms, but that financial openness is related negatively to growth when the de-facto dimension is considered. We find mixed results for the trade openness dimensions based on models (9) and (10). While the trade-to-GDP variable correlates positively with growth but is statistically insignificant, the Tariff_WITS coefficient is negative when we use the FD-specification. LMF_open has a negative sign, but it is only significant in model (9). KAOPEN shows positive correlation coefficients, which are, however, linked to large standard errors.

**Table 9:** Statistical inference based on clustered (heteroskedasticity-robust) standard errors

	FD	5-year av	FD	5-year av	FD	5-year av	FD	5-year av	FD	5-year av
	(1)	(2)	(3)	(4)	(5)	(6)	(7)	(8)	(9)	(10)
*Dependent variable: GDP per capita growth*										
log(KOF_econ)	2.465	1.191								
	(2.701)	(1.077)								
log(KOF_trade)			6.240***	1.516*						
			(2.258)	(0.881)						
log(KOF_finance)			− 3.707*	− 0.058						
			(2.084)	(0.742)						
log(KOF_defacto)					− 0.651	0.068				
					(1.938)	(0.896)				
log(KOF_dejure)					5.492***	1.253				
					(1.973)	(0.844)				
log(KOF_trade_df)							4.297**	0.331		
							(1.726)	(0.749)		
log(KOF_trade_dj)							2.569**	0.989*		
							(1.295)	(0.558)		
log(KOF_finance_df)							− 4.739***	− 0.056		
							(1.776)	(0.629)		
log(KOF_finance_dj)							1.280	0.277		
							(1.772)	(0.517)		
log(Trade_to_GDP)									5.221	0.366
									(3.245)	(0.601)
log(Tariff_WITS)									− 8.872	0.392
									(5.567)	(1.209)
log(LMF_open)									− 8.179***	− 0.556
									(2.104)	(0.458)
log(KAOPEN)									0.421	0.006
									(0.392)	(0.333)
log(hc)	6.018	0.124	8.715	0.161	4.295	− 0.014	9.459	− 0.150	− 24.647	3.438
	(8.634)	(1.695)	(9.543)	(1.668)	(8.111)	(1.659)	(9.616)	(1.708)	(16.191)	(3.471)
pop_growth	− 0.565**	− 0.512**	− 0.565**	− 0.495**	− 0.566**	− 0.503**	− 0.564**	− 0.490**	− 0.820*	− 0.598***
	(0.276)	(0.203)	(0.270)	(0.204)	(0.279)	(0.201)	(0.277)	(0.201)	(0.471)	(0.193)
Inflation	0.001	− 0.002***	0.001	− 0.002***	0.001	− 0.002****	0.001	− 0.002***	0.001***	− 0.001***
	(0.001)	(0.001)	(0.001)	(0.001)	(0.001)	(0.001)	(0.001)	(0.001)	(0.0001)	(0.0001)
log(inv_share)	− 0.058	1.503***	− 0.241	1.433**	− 0.055	1.469***	− 0.381	1.415**	0.559	0.639
	(1.460)	(0.558)	(1.457)	(0.575)	(1.466)	(0.552)	(1.464)	(0.570)	(2.386)	(1.285)
Constant	− 0.103		− 0.106		− 0.105		− 0.094		0.291*	
	(0.098)		(0.106)		(0.095)		(0.110)		(0.171)	
Observations	5127	1179	5127	1179	4102	1173	5096	1172	1807	460
R^2^	0.002	0.066	0.005	0.068	0.004	0.067	0.007	0.069	0.046	0.033
F Statistic	2.187* (df = 5; 5121)	14.561*** (df = 5; 1033)	4.045*** (df = 6; 5120)	12.522*** (df = 6; 1032)	3.093*** (df = 6; 5095)	12.302*** (df = 6; 1027)	4.656*** (df = 8; 5087)	9.436*** (df = 8; 1024	10.807*** (df = 8; 1798)	1.424 (df = 8; 334)

Country- and time-fixed effects were included. When running the specifications in first differences, however, the country-fixed effects drop out algebraically

*, **, and *** denote statistical significance at the 10%, 5% and 1% level, respectively

While we do not claim that we provide a fully-fledged estimation framework or that we show a definite answer on the relationship between economic openness and growth—both of which would require a much more careful consideration of possible endogeneity and reverse causality issues –, we can nevertheless use the standard regression framework to derive some general conclusions on the use of openness indicators. Our results indicate that operationalizing economic openness for econometric research requires explicit theoretical justifications of the relevant dimensions as well as the available indicators within these dimensions. Differences in how openness indicators correlate with economic growth illustrate the theory-laddenness of observation (Hanson 1958), i.e. the assumptions underlying the construction of different openness indicators make an important difference. At the same time, specifying growth regressions with more than one openness indicator, or running extensive robustness checks with different indicators, can provide hints regarding how different types of economic openness relate to GDP growth or other variables of interest.

## Conclusions

This paper has reviewed existing measures and empirical practices regarding economic openness, which we can generally understand as the degree to which non-domestic actors can or do participate in the domestic economy. We have compiled openness indicators by merging publicly available data from different sources—the data set is published together with this article—and have categorized the indicators using a typology of economic openness, which distinguishes between ‘real’ and ‘financial’ openness, as well as a ‘de-facto’ dimension (based on aggregate economic statistics) and a ‘de-jure’ dimension (focusing on institutional foundations of openness), respectively. The data set consists of 216 countries over the time period 1965–2019, although there is wide variation in the coverage of the country and time dimension across different openness variables.

We have used this data set to analyze the correlation across indicators, both in levels and in first differences. We find that indicators that belong to the same category of openness measures indeed tend to be correlated more strongly. Correlations among openness indicators are, however, in general much weaker in the case of first differences. By using a standard growth regression framework, we have shown how different types of economic openness as well as different indicators capture the impact of openness on economic growth in different ways. From this finding, it follows that applied researchers are well advised to motivate their choice of openness indicator rigorously, since different research questions might also entail different conceptions of economic openness. At the same time, it can be argued that the identification of reasons for why different measures of economic openness yield different results is an important and rewarding research activity.

## Electronic supplementary material

The data and code files required to replicate the results presented in this paper are available on GitHub: https://github.com/graebnerc/econ-openness. The R package for downloading the data on all the economic openness variables used in this paper is also available on GitHub: https://github.com/graebnerc/OpennessDataR. Below is the link to the electronic supplementary material.

Supplementary material 1 (PDF 476 kb)

## Footnotes

1 The package is available via Github: https://github.com/graebnerc/OpennessDataR. Researchers who do not use R can nevertheless download the fully compiled data, which will be regularly updated by the authors. The code and data files required to reproduce all the findings of this paper are available via Github as well: https://github.com/graebnerc/econ-openness.

2 For example, Frankel and Romer (2000) prominently use predictions from a gravity equation to instrument for trade openness in cross-sectional regressions. Felbermayr and Gröschl (2013) estimate the effects of (instrumented) trade openness on income levels with panel data.

3 One might be tempted to correct for such specific characteristics within the openness measure itself, but the problem with such practice would be that it sorts out one particular determinant of trade intensity in a potentially ad hoc manner. For example, since remoteness obviously impacts trade flows, a de-facto measure for openness should, ceteris paribus, be lower for more remote countries. There might be cases in which measures correcting for remoteness are useful, but they need to be carefully justified against the specific background of a given application and are, thus, not considered further in this general review.

4 A common reaction in the literature to address the problem of business cycles has been the use of 5-year averages, a practice that comes with a number of other problems as discussed in Herzer and Vollmer ().

5 The latest LMF dataset is available here: https://www.imf.org/en/Publications/WP/Issues/2017/05/10/International-Financial-Integration-in-the-Aftermath-of-the-Global-Financial-Crisis-44906  [last download on August 21st 2020].

6 Existing differences between the FDI time series provided by Lane and Milesi-Ferretti (2017) in comparison to UNCTAD (2017) can be traced back to a partly different usage of balance of payment manuals: for some countries, the two sources treat reverse investment (between affiliates and parent companies) differently, which leads to deviations in the reported FDI assets and liabilities.

7 The IMF’s AREAER report draws on information from official sources and has been prepared in close consultation with national authorities. For more information visit: https://www.imf.org/~/media/Files/Publications/AREAER/AREAER_2016_Overview.ashx [last download on August 21st 2020].

8 Note that the covered time period is shorter for some countries due to data availability.

9 The Chinn-Ito-Index has been criticized for measuring more the *extensity* than the *intensity* of capital controls. In response, Chinn and Ito (2008) compare their index with de-jure indices that focus on the intensity of capital controls (e.g. CAPITAL in Table 6) and find a high correlation between CAPITAL and KAOPEN suggesting that KAOPEN is a valid proxy for the *intensity* of capital controls.

10 The index of economic complexity (ECI) infers the technological capabilities of an economy by considering the products, in which countries have a revealed comparative advantage. It starts from the empirical observation that most developed and technologically advanced countries export a great diversity of products and argues that rare products within these diversified export baskets are associated with a high degree of complexity. In contrast, less technologically developed countries typically show a much smaller degree of diversification in their export basket—as a consequence, their revealed comparative advantage often lies in low labor costs or specific natural resources, which come with a low degree of technological complexity. For a detailed description of the methodology see Hidalgo and Hausmann (2009).

11 The classification into complexity groups and basic information on the data are provided in detail in the Online Appendix.

12 Unit roots tests for the individual time series are provided in the appendix. The Sachs-Warner as an index is excluded from this analysis.

13 Notably, we use annual data (and not 5-year averages as in Eq. (1)) to estimate the first difference specification in Eq. (2).
