# Supplementary material for: Understanding economic openness: a review of existing measures
Source: Rev World Econ. 2020 Sep 1;157(1):87–120. doi: 10.1007/s10290-020-00391-1 (PMC7461754; doi:10.1007/s10290-020-00391-1)
Supplement: Supplementary file 1 — Supplementary material 1 (PDF 476 kb) [file 10290_2020_391_MOESM1_ESM.pdf]

Appendix: Understanding economic openness

A review of existing measures

Supplementary material*

Abstract

We provide the descriptive statistics for all data used in the paper in section A. In section B we describe how we grouped countries for the analysis in section 3 in the main paper, and provide for the figures with countries grouped according to their level of income (section C). We then rank countries according to their openness in selected indicators, as well as the discrepancy between their *de facto* and *de jure* openness in section D. In section E we replicate the correlation analysis of section 4 in the main paper using the Pearson instead of the Spearman correlation coefficient. Finally, in section F we provide more detailed information about the regression results underlying table 8 in the main paper.

Contents

A Descriptive statistics and country set **2**

A.1 The full data set 2

A.2 The reduced data set 7

B Country groups according to economic complexity **9**

C Trends in openness based on income groups **9**

D Rankings **12**

E Correlation analysis with alternative correlation measures **15**

F More detailed regression results **17**

*The authors acknowledge funds of the Oesterreichische Nationalbank (OeNB, Anniversary Fund, project number: 17383).

A Descriptive statistics and country set

A.1 The full data set

Table 1 provides the descriptive statistics for the variables used in the paper.[1]

Table 1: Descriptive statistics for all indicators included in the data set.

Indicator	Observations	Countries	Year_min	Year_max
Alcala	5446	173	1960	2014
CAPITAL	3858	95	1960	2004
chinn_ito_normed	7235	181	1970	2017
CTS	7090	231	1977	2016
EXP_to_GDP	8322	199	1960	2018
Exports_USD_constant	6383	189	1960	2018
Exports_USD_current	8265	198	1960	2018
FIN_CUR	3858	95	1960	2004
FTI_original	2514	161	1995	2017
FTI_original_ipo	2718	161	1995	2017
FTI_panel	3125	162	1970	2017
FTI_reduced	2522	161	1995	2017
FTI_reduced_ipo	2730	161	1995	2017
GDP_pc_growth	8956	180	1960	2017
hc	7656	144	1960	2017
HF_econ	4043	181	1995	2019
HF_fin	4071	182	1995	2019
HF_trade	4062	182	1995	2019
IMP_to_GDP	8331	199	1960	2018
Imports_USD_constant	6383	189	1960	2018
Imports_USD_current	8274	198	1960	2018
inflation	7711	185	1960	2018
inv_share	9224	180	1960	2017
KAOPEN	7235	181	1970	2017
KOF_defacto	8933	204	1970	2017
KOF_dejure	8553	193	1970	2017
KOF_econ	8841	201	1970	2017
KOF_finance	8901	202	1970	2017
KOF_finance_df	9032	204	1970	2017
KOF_finance_dj	8768	198	1970	2017
KOF_trade	8861	200	1970	2017
KOF_trade_df	9128	206	1970	2017
KOF_trade_dj	8291	193	1970	2017
Lietal	7441	233	1960	2016
LMF_EQ	7393	200	1970	2015
LMF_EQ_gdp	7375	200	1970	2015
LMF_FDI_total_stocks	7543	202	1970	2015
LMF_FDI_total_stocks_GDP	7525	202	1970	2015
LMF_open	7564	203	1970	2015
LMF_open_gdp	7545	203	1970	2015

[1]The data, as well as the code to reproduce the estimation results and figures will be available online after publication: [github link blinded for review]. Moreover, we provide an R package that allows one to automatically download the most recent versions of the indicators from the internet.

LMF_open_pv	6406	179	1970	2014
Penn_GDP_PPP	9224	180	1960	2017
Penn_GDP_PPP_log	9224	180	1960	2017
pop_growth	9119	180	1960	2017
pop_log	9224	180	1960	2017
population	12577	215	1960	2018
rgdpo	9224	180	1960	2017
Tariff_RES	3057	136	1980	2005
Tariff_WITS	2316	159	1988	2018
Tariff_WITS_ipo	2860	159	1988	2018
TOI	7079	167	1960	2016
Trade_to_GDP	8322	199	1960	2018
UNC_FDI_in_stock_GDP	6646	197	1980	2018
UNC_FDI_out_stock_GDP	4891	174	1980	2018
UNC_FDI_total_stocks_GDP	4839	174	1980	2018

Table 2 provides information for all countries present in the data set, as well as the total number of available observations for each country.

Table 2: Countries included in the full data set.

Country	Observations	Year_min	Year_max
Aruba	1187	1960	2018
Afghanistan	918	1960	2019
Angola	1625	1960	2019
Albania	1758	1960	2019
Andorra	140	1960	2018
United Arab Emirates	1715	1960	2019
Argentina	2424	1960	2019
Armenia	1278	1960	2019
American Samoa	178	1960	2018
Antigua & Barbuda	1454	1960	2018
Australia	2452	1960	2019
Austria	2281	1960	2019
Azerbaijan	1235	1960	2019
Burundi	2182	1960	2019
Belgium	2273	1960	2019
Benin	2272	1960	2019
Burkina Faso	2172	1960	2019
Bangladesh	2303	1960	2019
Bulgaria	1877	1960	2019
Bahrain	2063	1960	2019
Bahamas	1984	1960	2019
Bosnia & Herzegovina	1095	1960	2019
Belarus	1234	1960	2019
Belize	1835	1960	2019
Bermuda	896	1960	2018
Bolivia	2470	1960	2019
Brazil	2475	1960	2019
Barbados	2010	1960	2019
Brunei	1471	1960	2019

Bhutan	1566	1960	2019
Botswana	2350	1960	2019
Central African Republic	1830	1960	2019
Canada	2510	1960	2019
Switzerland	2202	1960	2019
Chile	2481	1960	2019
China	2179	1960	2019
Côte d'Ivoire	2206	1960	2019
Cameroon	2328	1960	2019
Congo - Kinshasa	1471	1960	2019
Congo - Brazzaville	2363	1960	2019
Colombia	2508	1960	2019
Comoros	1589	1960	2019
Cape Verde	1721	1960	2019
Costa Rica	2468	1960	2019
Cuba	541	1960	2019
Curaçao	258	1960	2018
Cayman Islands	748	1960	2018
Cyprus	2149	1960	2019
Czechia	1365	1960	2019
Germany	2377	1960	2019
Djibouti	1252	1960	2019
Dominica	1637	1960	2019
Denmark	2394	1960	2019
Dominican Republic	2385	1960	2019
Algeria	2393	1960	2019
Ecuador	2418	1960	2019
Egypt	2469	1960	2019
Eritrea	627	1960	2019
Spain	2369	1960	2019
Estonia	1314	1960	2019
Ethiopia	1843	1960	2019
Finland	2371	1960	2019
Fiji	1856	1960	2019
France	2439	1960	2019
Faroe Islands	323	1960	2018
Micronesia (Federated States of)	581	1960	2018
Gabon	2350	1960	2019
United Kingdom	2379	1960	2019
Georgia	1190	1960	2019
Ghana	2269	1960	2019
Gibraltar	140	1960	2018
Guinea	1827	1960	2019
Gambia	2041	1960	2019
Guinea-Bissau	1801	1960	2019
Equatorial Guinea	1489	1960	2019
Greece	2390	1960	2019
Grenada	1599	1960	2018
Greenland	215	1960	2018
Guatemala	2443	1960	2019
Guam	226	1960	2018
Guyana	1586	1960	2019
Hong Kong SAR China	2318	1960	2019

Honduras	2406	1960	2019
Croatia	1289	1960	2019
Haiti	2104	1960	2019
Hungary	1876	1960	2019
Indonesia	2452	1960	2019
Isle of Man	133	1960	2018
India	2490	1960	2019
Ireland	2261	1960	2019
Iran	2210	1960	2019
Iraq	1468	1960	2018
Iceland	2351	1960	2019
Israel	2436	1960	2019
Italy	2379	1960	2019
Jamaica	2326	1960	2019
Jordan	2341	1960	2019
Japan	2370	1960	2019
Kazakhstan	1273	1960	2019
Kenya	2454	1960	2019
Kyrgyzstan	1297	1960	2019
Cambodia	1692	1960	2019
Kiribati	980	1960	2019
St. Kitts & Nevis	1455	1960	2018
South Korea	2445	1960	2019
Kuwait	2047	1960	2019
Laos	1768	1960	2019
Lebanon	1783	1960	2019
Liberia	1814	1960	2019
Libya	1348	1960	2019
St. Lucia	1567	1960	2019
Liechtenstein	200	1960	2019
Sri Lanka	2461	1960	2019
Lesotho	2065	1960	2019
Lithuania	1297	1960	2019
Luxembourg	1877	1960	2019
Latvia	1297	1960	2019
Macau SAR China	1477	1960	2019
Saint Martin (French part)	59	1960	2018
Morocco	2406	1960	2019
Monaco	59	1960	2018
Moldova	1278	1960	2019
Madagascar	2358	1960	2019
Maldives	1584	1960	2019
Mexico	2468	1960	2019
Marshall Islands	629	1960	2018
Macedonia	1266	1960	2019
Mali	2290	1960	2019
Malta	2134	1960	2019
Myanmar (Burma)	1843	1960	2019
Montenegro	1102	1960	2019
Mongolia	1687	1960	2019
Northern Mariana Islands	178	1960	2018
Mozambique	1823	1960	2019
Mauritania	2242	1960	2019

Mauritius	2285	1960	2019
Malawi	2187	1960	2019
Malaysia	2489	1960	2019
Namibia	1737	1960	2019
New Caledonia	422	1960	2018
Niger	2128	1960	2019
Nigeria	2394	1960	2019
Nicaragua	2334	1960	2019
Netherlands	2377	1960	2019
Norway	2417	1960	2019
Nepal	2210	1960	2019
Nauru	171	1960	2018
New Zealand	2327	1960	2019
Oman	1970	1960	2019
Pakistan	2421	1960	2019
Panama	2352	1960	2019
Peru	2440	1960	2019
Philippines	2440	1960	2019
Palau	519	1960	2018
Papua New Guinea	1473	1960	2019
Poland	1903	1960	2019
Puerto Rico	319	1960	2018
North Korea	165	1960	2019
Portugal	2359	1960	2019
Paraguay	2379	1960	2019
Palestinian Territories	823	1970	2018
French Polynesia	326	1960	2018
Qatar	1731	1960	2019
Romania	1434	1960	2019
Russia	1413	1960	2019
Rwanda	2241	1960	2019
Saudi Arabia	2064	1960	2019
Sudan	2095	1960	2019
Senegal	2442	1960	2019
Singapore	2496	1960	2019
Solomon Islands	1156	1960	2019
Sierra Leone	2217	1960	2019
El Salvador	2423	1960	2019
San Marino	321	1960	2018
Somalia	765	1960	2018
Serbia	1182	1970	2019
South Sudan	138	1960	2018
São Tomé & Príncipe	1218	1960	2019
Suriname	1428	1960	2019
Slovakia	1315	1960	2019
Slovenia	1344	1960	2019
Sweden	2449	1960	2019
Swaziland	2021	1960	2018
Sint Maarten	193	1998	2018
Seychelles	1843	1960	2019
Syria	2037	1960	2019
Turks & Caicos Islands	524	1960	2018
Chad	2039	1960	2019

Togo	2282	1960	2019
Thailand	2484	1960	2019
Tajikistan	1098	1960	2019
Turkmenistan	858	1960	2019
Timor-Leste	381	1960	2019
Tonga	1247	1960	2019
Trinidad & Tobago	1972	1960	2019
Tunisia	2396	1960	2019
Turkey	2406	1960	2019
Tuvalu	193	1960	2018
Taiwan	1014	1960	2019
Tanzania	2097	1960	2019
Uganda	2305	1960	2019
Ukraine	1352	1960	2019
Uruguay	2465	1960	2019
United States	2434	1960	2019
Uzbekistan	969	1960	2019
St. Vincent & Grenadines	1562	1960	2019
Venezuela	2388	1960	2019
British Virgin Islands	663	1960	2018
U.S. Virgin Islands	171	1960	2018
Vietnam	1744	1960	2019
Vanuatu	1118	1960	2019
Samoa	1209	1960	2019
Yemen	1211	1960	2019
South Africa	2518	1960	2019
Zambia	2087	1960	2019
Zimbabwe	1993	1960	2019

A.2 The reduced data set

Table 3 provide more information about the reduced data set used for the regressions in the main paper.

Table 3: The countries used in the regressions of the main paper.

Country	Observations
Albania	11
Armenia	11
Australia	20
Benin	11
Burkina Faso	10
Bangladesh	11
Bahrain	15
Bolivia	12
Brazil	19
Botswana	14
Canada	20
Chile	20
China	15
Côte d'Ivoire	10

Cameroon	15
Congo - Brazzaville	8
Colombia	20
Costa Rica	15
Algeria	10
Egypt	20
Guatemala	15
Hong Kong SAR China	17
Honduras	11
Croatia	13
Indonesia	15
India	20
Iceland	18
Israel	16
Jamaica	10
Jordan	15
Japan	19
Kenya	15
South Korea	20
Kuwait	10
Sri Lanka	15
Morocco	13
Moldova	10
Madagascar	15
Mexico	20
Mali	15
Mongolia	11
Mauritius	15
Malawi	13
Malaysia	20
Namibia	12
Nigeria	11
Nicaragua	10
Norway	19
New Zealand	15
Pakistan	12
Peru	20
Philippines	19
Russia	19
Senegal	13
Singapore	19
El Salvador	14
Thailand	20
Tunisia	15
Turkey	20
Ukraine	15
Uruguay	15
United States	20
Venezuela	5
Vietnam	10
South Africa	20
Total	971

B Country groups according to economic complexity

We classified countries according to their complexity as defined by Hidalgo and Hausmann. We decided to set thresholds such that the three groups (*high*, *medium*, and *low* complexity) consist of approximately the same number of countries. This yields to the following classification, according to which we classify countries every year anew (i.e. countries can in principle switch between groups):

High complexity	$ECI > 0.5$
Medium complexity	$0.5 \geq ECI \geq -0.5$
Low complexity	$ECI < -0.5$

C Trends in openness based on income groups

In the main paper we classified countries according to their complexity as defined by Hidalgo and Hausmann and as explicated in section B. Here we complement this presentation by providing the same kind of visualization, but according to the income groups as provided by the World Bank. The World Bank assigns countries into four income groups – high, upper-middle, lower-middle, and low. The assignment is based on the GNI per capita in current US dollars calculated using the Atlas method. The threshold levels are determined at the start of the Bank's fiscal year in July and remain fixed for 12 months regardless of subsequent revisions to estimates. Thus, as for the classification into complexity groups, countries may move among income groups over the years. Currently, the following classification scheme is used:

	GNI p.c. in current USD
High income	> 12235
Upper middle income	$3956 - 12235$
Lower middle income	$1006 - 3955$
Low income	< 1005

The figures of section 3 in the main text are replicated in figures 1 (for figure 1 in the main text), 2 (for figure 2 in the main text), and 3 (for figure 3 in the main text) using the World Bank classification. Note that since our sample is restricted to European countries only high and upper medium income countries show up.

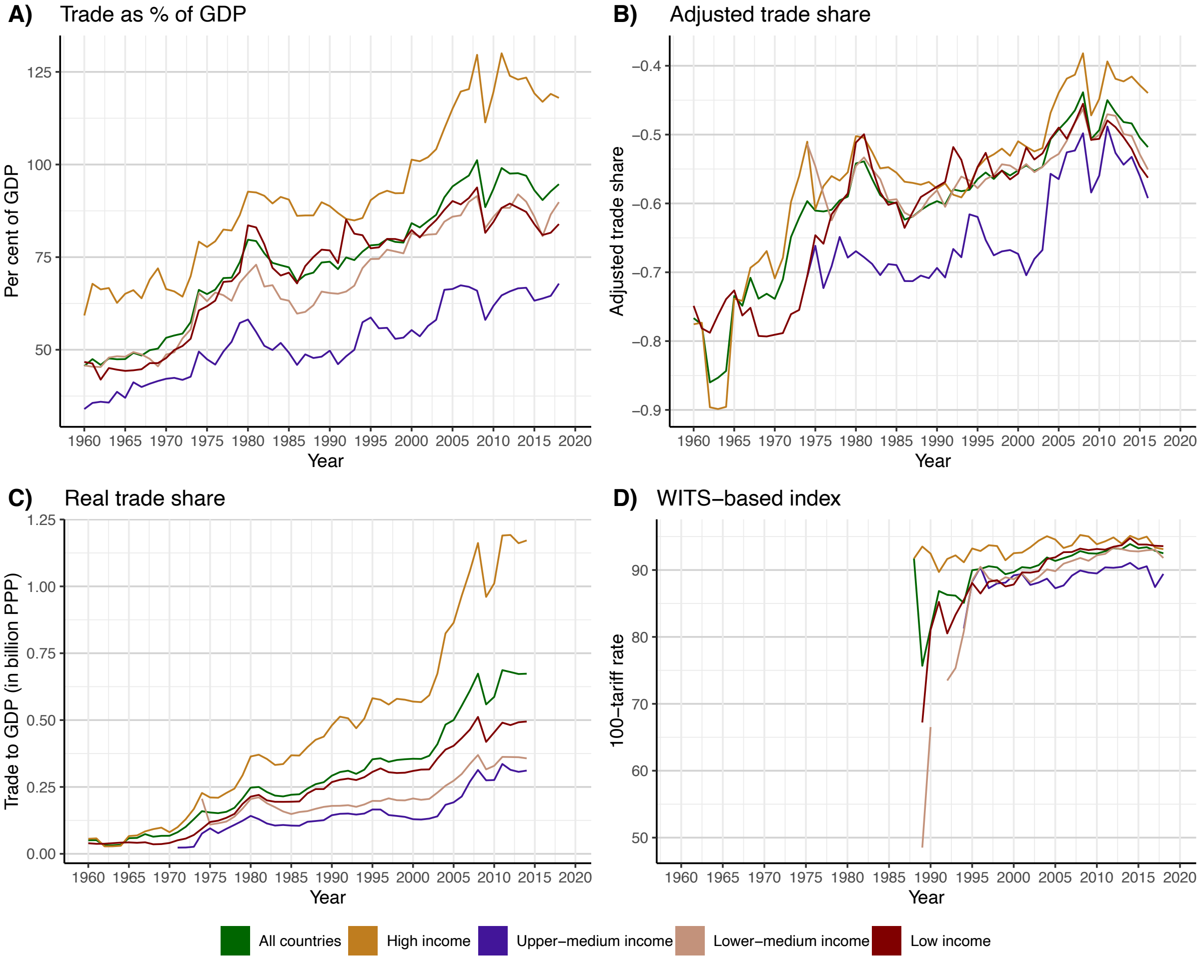

Figure 1: Replication of figure 1 in the main text: the dynamics of trade openness measures.

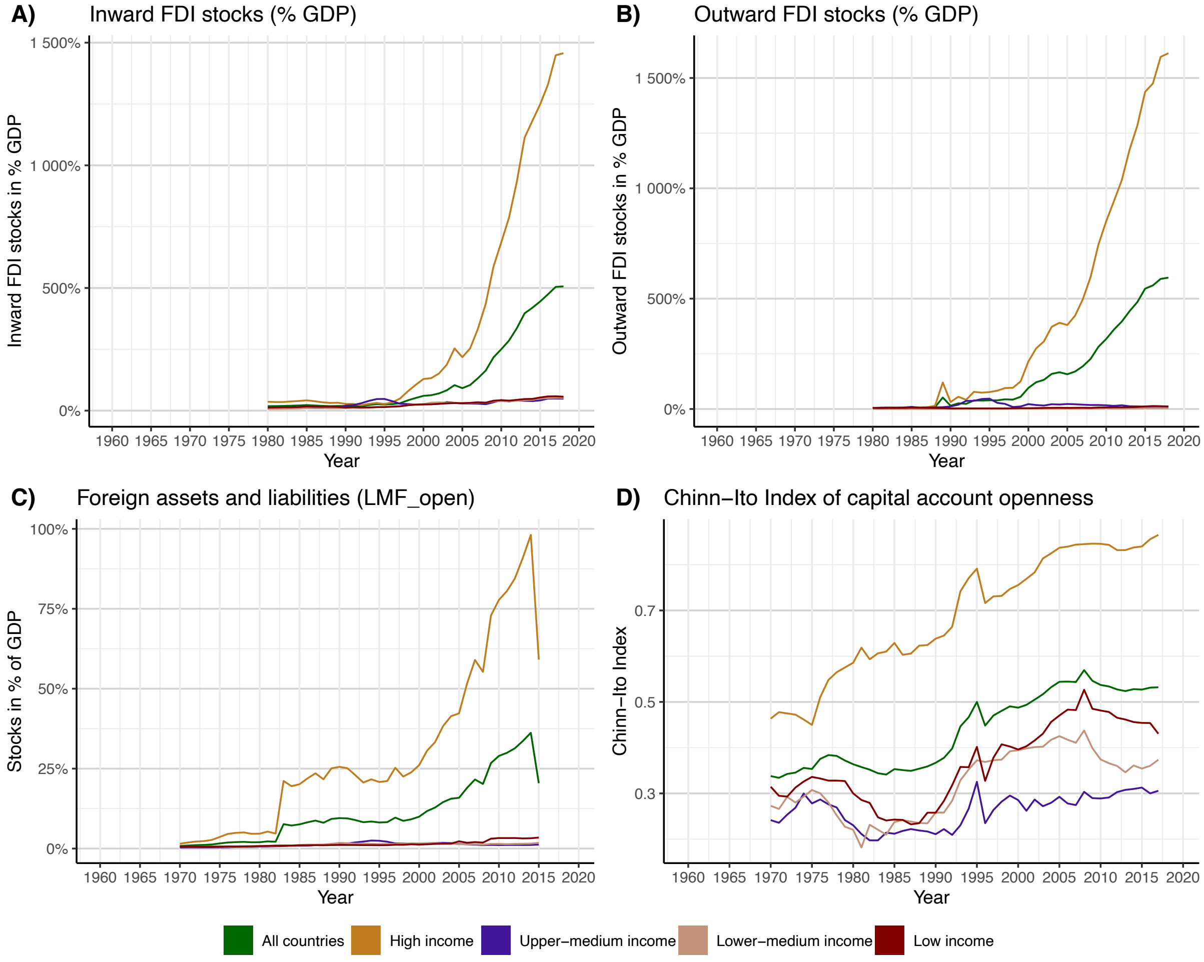

Figure 2: Replication of figure 2 in the main text: the dynamics of financial openness measures.

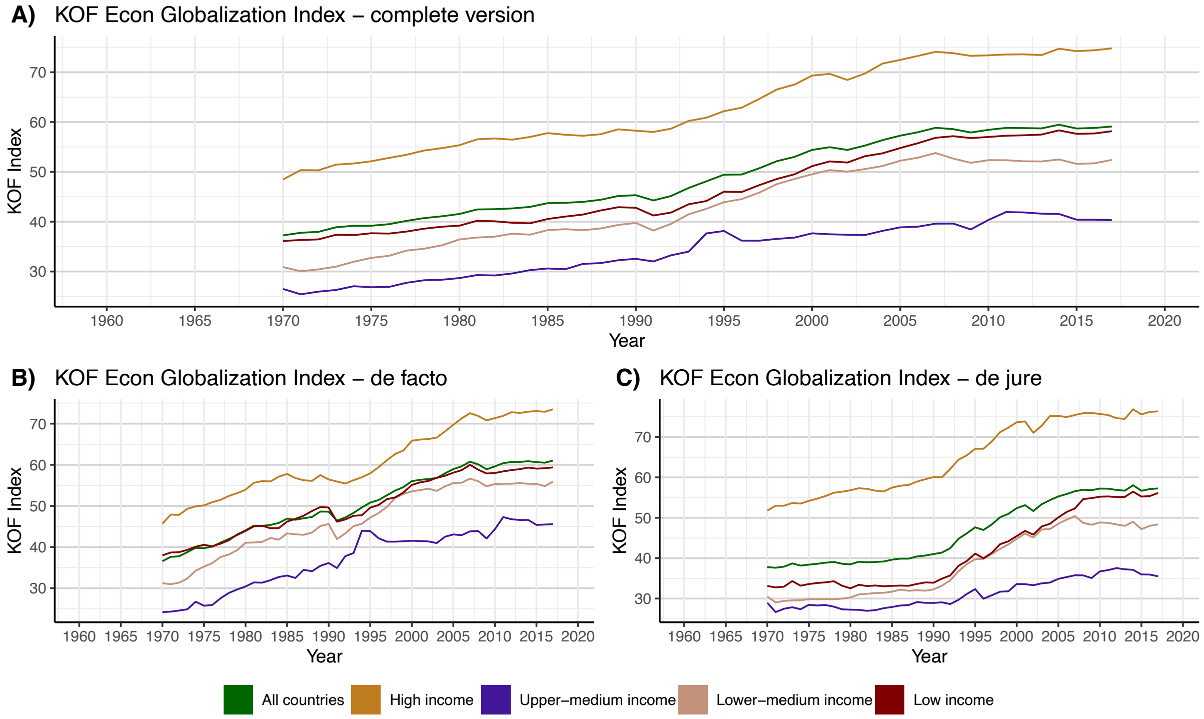

Figure 3: Replication of figure 3 in the main text: the dynamics of the KOF hybrid measure.

D Rankings

Here we first rank countries according to selected openness measures (see table 4) and, second, illustrate the fact that a high degree of *de jure* openness does not necessarily implies a high degree of *de facto* openness: figure 4 illustrates this difference and highlights those countries with the strongest discrepancy between *de facto* and *de jure* openness.

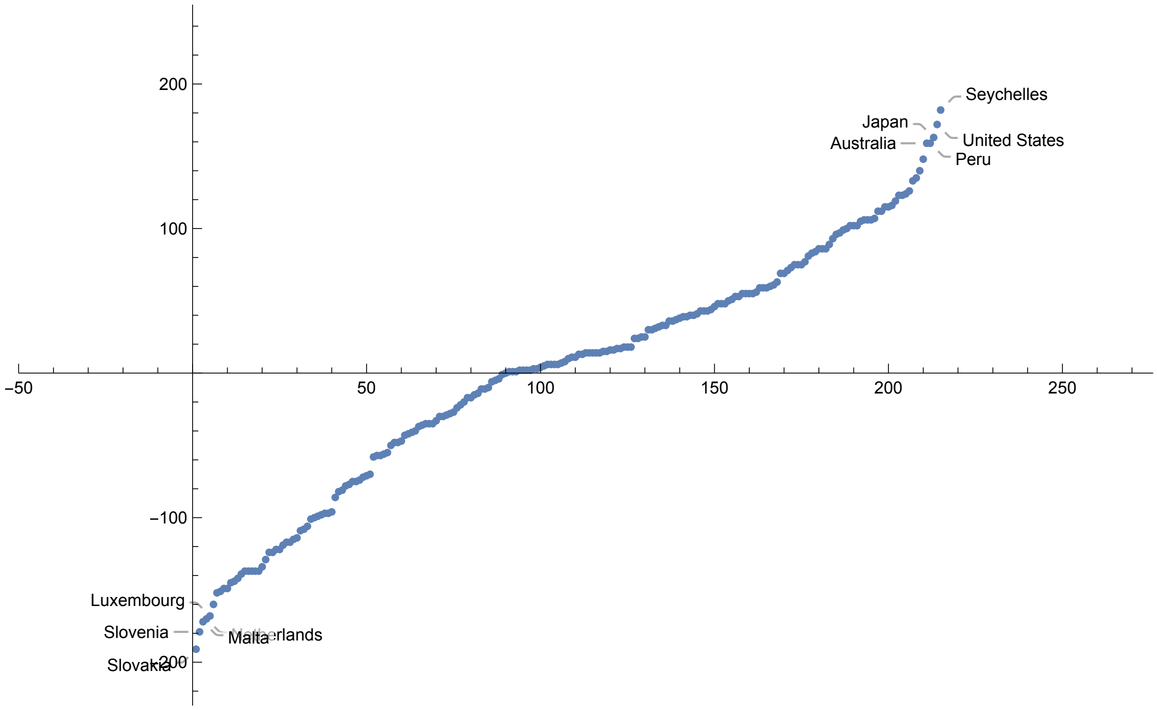

(a) Differences in the ranks of trade-to-GDP (trade *de facto*) and the WITS-based index (trade *de jure*).

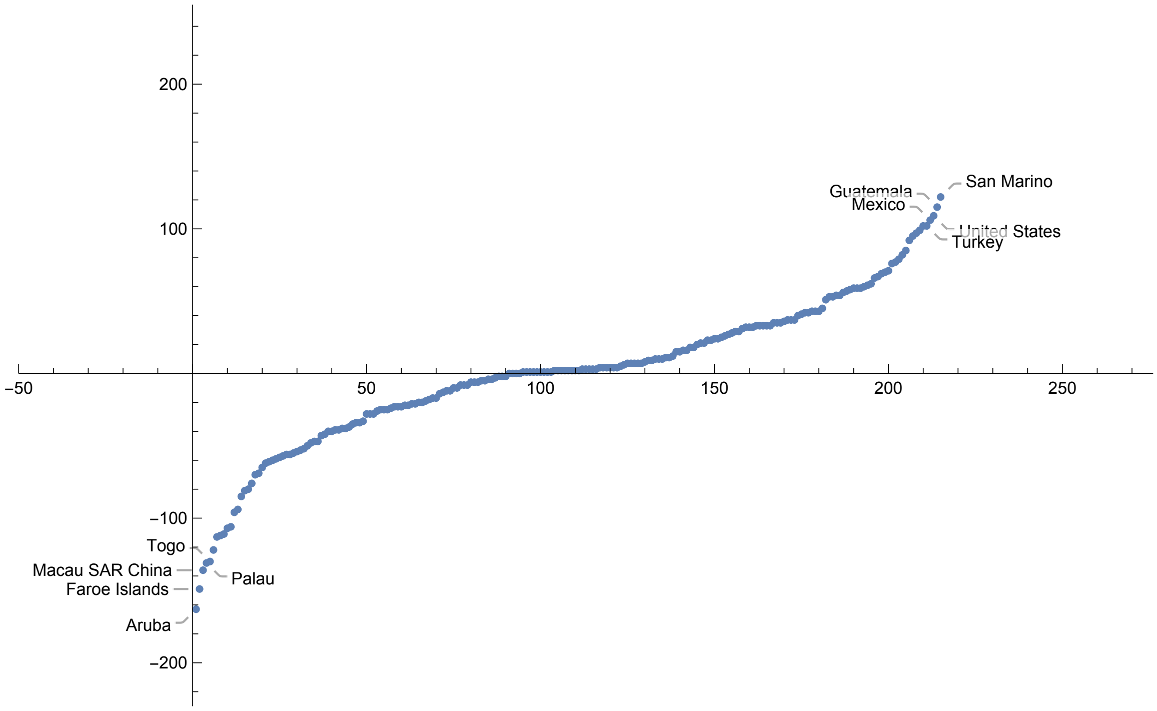

(b) Differences in the ranks of KOF *de facto* and KOF *de jure*.

Figure 4: Comparisons of *de facto* and *de jure* openness.

Country	Rank
Luxembourg	1
Hong Kong SAR China	2
Singapore	3
Malta	4
Djibouti	5
Sint Maarten	6
Ireland	7
Slovakia	8
Vietnam	9
United Arab Emirates	10
San Marino	206
São Tomé & Príncipe	207
Suriname	208
Syria	209
Turks & Caicos Islands	210
Trinidad & Tobago	211
Tuvalu	212
Taiwan	213
British Virgin Islands	214
Vanuatu	215
Yemen	216

(a) Rank according to trade-to-GDP (trade *de facto*).

Country	Rank
Hong Kong SAR China	2
Macau SAR China	2
Singapore	3
Mauritius	4
Georgia	5
Peru	6
New Zealand	7
Switzerland	8
Ukraine	9
United States	10
Turkmenistan	206
Timor-Leste	207
Tonga	208
Trinidad & Tobago	209
Tuvalu	210
Taiwan	211
Uzbekistan	212
Venezuela	213
British Virgin Islands	214
U.S. Virgin Islands	215
Vanuatu	216

(b) Rank according to the WITS-based index (trade *de jure*).

Country	Rank
Singapore	1
Netherlands	2
Malta	3
Hong Kong SAR China	4
United Arab Emirates	5
Belgium	6
Bahrain	7
Ireland	8
Mauritius	9
Seychelles	10
Romania	206
San Marino	207
Somalia	208
South Sudan	209
Sint Maarten	210
Turks & Caicos Islands	211
Timor-Leste	212
Tuvalu	213
Taiwan	214
British Virgin Islands	215
U.S. Virgin Islands	216

(c) Rank according to the KOF *de facto* index.

Country	Rank
Luxembourg	1
Singapore	2
Ireland	3
United Kingdom	4
Czechia	5
Finland	6
Sweden	7
Estonia	8
Netherlands	9
Belgium	10
Romania	206
Somalia	207
South Sudan	208
Sint Maarten	209
Turks & Caicos Islands	210
Turkmenistan	211
Timor-Leste	212
Tuvalu	213
Taiwan	214
British Virgin Islands	215
U.S. Virgin Islands	216

(d) Rank according to the KOF *de jure* index.

Table 4: The most and least open countries according to selected openness measures.

E Correlation analysis with alternative correlation measures

Here we replicate the correlation matrix of section 4 in the main paper with the Pearson correlation coefficient (see figure 5 for correlations among levels and 6 for correlations among differences). The assumptions for this measure are somehow more restrictive than for the Spearman coefficient, yet the results are more pronounced, and the clusters of trade vs. financial, and de facto vs. de jure measures are easier to spot.

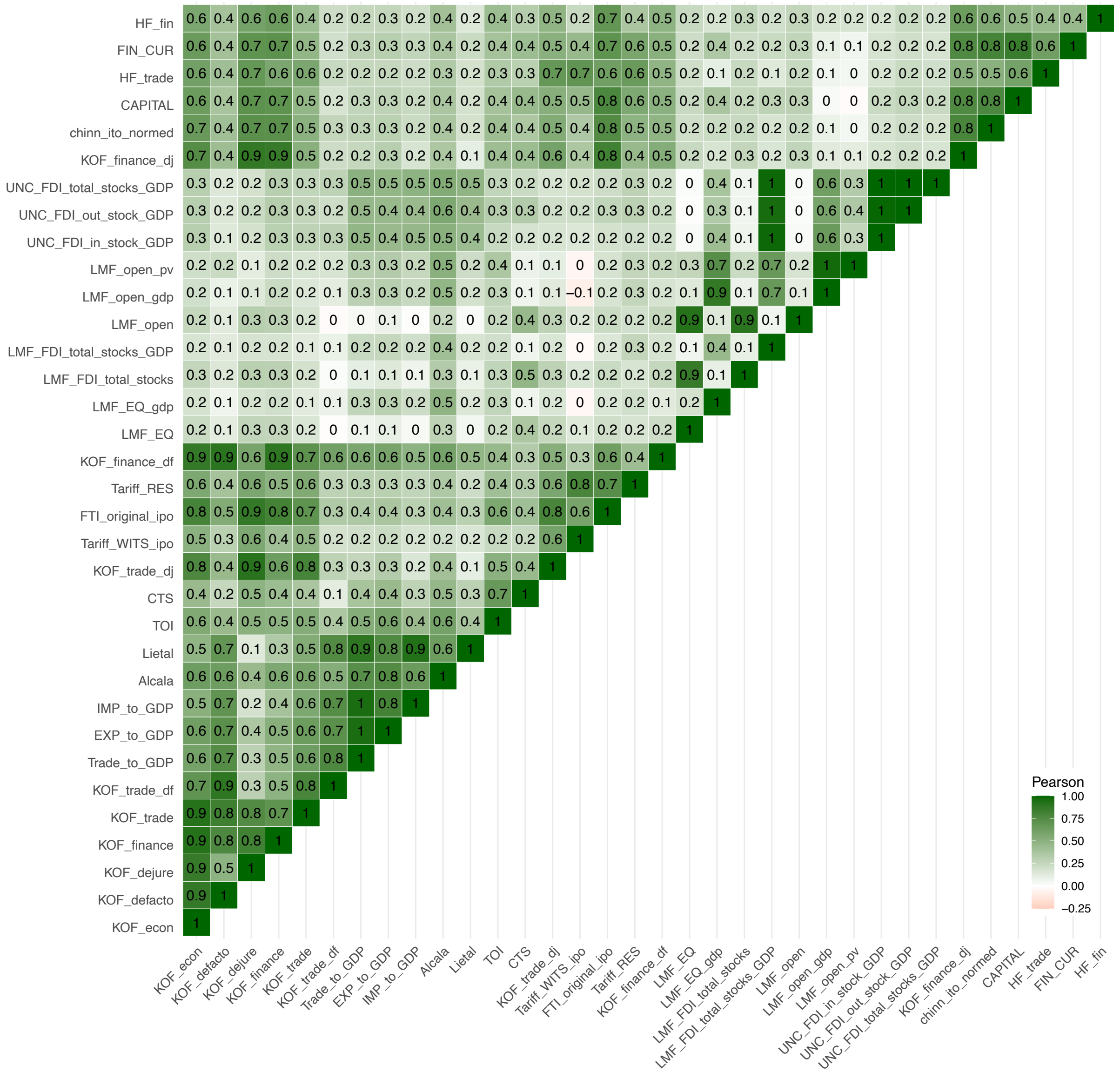

Figure 5: The correlation analysis for level data using the Pearson correlation coefficient.

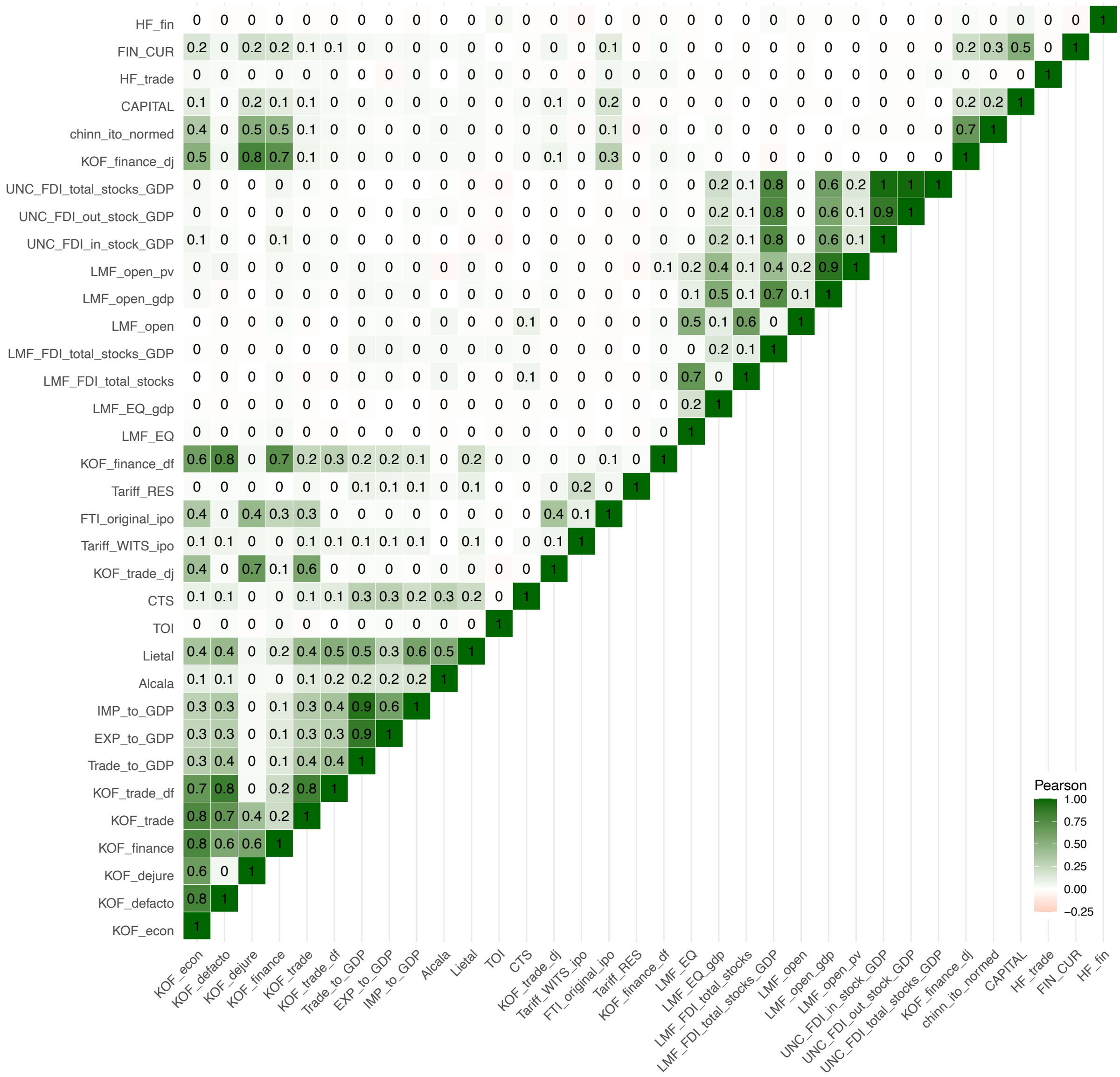

Figure 6: The correlation analysis for differenced data using the Pearson correlation coefficient.

F More detailed regression results

Here we provide the detailed results for the regressions summarized in table 7 in the main paper.

Table 5 provides the results for de facto trade openness measures, table 6 for de jure trade openness measures, table 7a for de facto financial openness measures, and, finally, table 7b for de jure financial openness measures.

	Dependent variable: GDP per capita growth					
	(1)	(2)	(3)	(4)	(5)	(6)
log(Trade_to_GDP)	0.777					
	(1.813)					
log(Alcala)		2.844^{***}				
		(0.974)				
log(Lietal)			1.512			
			(2.725)			
log(TOI)				−1.372		
				(2.293)		
log(KOF_defacto)					1.923	
					(0.875)	
log(CTS)						2.000
						(2.045)
log(initial_GDP_pc)	-7.036^{***}	-7.746^{***}	-6.994^{***}	-7.435^{***}	-7.094^{***}	-7.710^{***}
	(1.152)	(1.062)	(1.106)	(1.351)	(−6.114)	(−6.266)
log(hc)	22.138^{***}	15.880^{**}	21.508^{***}	23.016^{***}	21.323	20.511
	(5.591)	(6.139)	(5.574)	(5.322)	(3.725)	(3.854)
pop_growth	−0.335	−0.209	−0.359	−0.403	-0.325^{***}	-0.340^{***}
	(0.455)	(0.494)	(0.442)	(0.429)	(−0.721)	(−0.764)
inflation	0.006^{***}	0.006^{***}	0.006^{***}	0.006^{***}	0.006	0.006
	(0.001)	(0.001)	(0.001)	(0.001)	(5.078)	(5.570)
log(inv_share)	3.843^{***}	3.115^{***}	3.738^{***}	3.753^{***}	3.858	3.334
	(0.953)	(0.987)	(1.008)	(0.929)	(4.051)	(3.174)
Observations	269	269	269	269	269	269
R^2	0.242	0.278	0.243	0.243	0.245	0.268
F Statistic	10.219^{***}	12.302^{***}	10.300^{***}	10.251^{***}	10.408^{***}	11.708^{***}
Note:						*p<0.1; **p<0.05; ***p<0.01

Table 5: Detailed regression results for de facto trade openness measures.

	Dependent variable: GDP per capita growth			
	(1)	(2)	(3)	(4)
log(KOF_dejure)	3.399**			
	(1.538)			
log(Tariff_WITS_ipo)		19.811***		
		(6.568)		
log(FTI_original_ipo)			7.619	
			(4.827)	
log(HF_trade)				1.716
				(2.225)
log(initial_GDP_pc)	−7.321***	−7.463***	−6.962***	−7.378***
	(1.146)	(1.178)	(1.166)	(1.224)
log(hc)	20.983***	16.592***	21.152***	21.125***
	(5.364)	(6.086)	(5.537)	(5.993)
pop_growth	−0.185	−0.300	−0.368	−0.308
	(0.419)	(0.406)	(0.423)	(0.441)
inflation	0.006***	0.005***	0.006***	0.005***
	(0.001)	(0.001)	(0.001)	(0.001)
log(inv_share)	3.605***	3.683***	3.543***	3.444***
	(0.956)	(0.927)	(0.974)	(1.119)
Observations	269	269	269	268
R^2	0.254	0.273	0.252	0.244
F Statistic	10.891***	12.041***	10.801***	10.259***

Note: *p<0.1; **p<0.05; ***p<0.01

Table 6: Detailed regression results for de jure trade openness measures.

	Dependent variable: GDP per capita growth			
	(1)	(2)	(3)	(4)
log(LMF_open_gdp)	0.144			
	(0.497)			
log(LMF_EQ_gdp)		−0.087		
		(0.271)		
log(UNC_FDI_in_stock_GDP)			0.620	
			(0.509)	
log(UNC_FDI_out_stock_GDP)				0.278
				(0.361)
log(initial_GDP_pc)	−7.080***	−7.013***	−7.172***	−7.338***
	(1.159)	(1.160)	(1.050)	(1.228)
log(hc)	22.745***	23.594***	20.201***	21.887***
	(5.133)	(5.512)	(5.436)	(5.625)
pop_growth	−0.362	−0.378	−0.382	−0.353
	(0.445)	(0.438)	(0.506)	(0.449)
inflation	0.006***	0.006***	0.006*	0.006***
	(0.001)	(0.001)	(0.003)	(0.001)
log(inv_share)	3.907***	3.928***	3.777***	4.020***
	(0.891)	(0.958)	(1.070)	(0.888)
Observations	269	269	269	269
R^2	0.241	0.241	0.247	0.245
F Statistic	10.173***	10.186***	10.485***	10.408***
Note:				*p<0.1; **p<0.05; ***p<0.01

(a) Table 7a: Detailed regression results for de facto financial openness measures.

	Dependent variable: GDP per capita growth		
	(1)	(2)	(3)
log(chinn_ito_normed)	0.280		
	(0.797)		
log(HF_fin)		−1.570	
		(1.423)	
log(CAPITAL)			0.768
			(1.652)
log(initial_GDP_pc)	−7.063***	−7.097***	−17.246***
	(1.153)	(1.141)	(3.822)
log(hc)	22.960***	22.008***	42.094**
	(5.257)	(5.225)	(19.934)
pop_growth	−0.364	−0.353	−0.220
	(0.441)	(0.436)	(1.971)
inflation	0.006***	0.006***	0.006***
	(0.001)	(0.001)	(0.002)
log(inv_share)	3.820***	3.770***	4.464**
	(0.961)	(0.906)	(1.864)
Observations	269	269	99
R^2	0.241	0.250	0.473
F Statistic	10.188***	10.662***	6.871***
Note:			*p<0.1; **p<0.05; ***p<0.01

(b) Table 7b: Detailed regression results for de jure financial openness measures.
